# EBNA2-deleted Epstein-Barr virus (EBV) isolate, P3HR1, causes Hodgkin-like lymphomas and diffuse large B cell lymphomas with type II and Wp-restricted latency types in humanized mice

**DOI:** 10.1371/journal.ppat.1008590

**Published:** 2020-06-15

**Authors:** Chunrong Li, James C. Romero-Masters, Shane Huebner, Makoto Ohashi, Mitchell Hayes, Jillian A. Bristol, Scott E. Nelson, Mark R. Eichelberg, Nicholas Van Sciver, Erik A. Ranheim, Rona S. Scott, Eric C. Johannsen, Shannon C. Kenney

**Affiliations:** 1 Department of Oncology, School of Medicine and Public Health, University of Wisconsin-Madison, Madison, Wisconsin, United States of America; 2 Department of Pathology and Laboratory Medicine, School of Medicine and Public Health, University of Wisconsin-Madison, Madison, Wisconsin, United States of America; 3 Center for Molecular and Tumor Virology, LSU Health Sciences Center, Shreveport, Louisiana, United States of America; 4 Department of Medicine, School of Medicine and Public Health, University of Wisconsin-Madison, Madison, Wisconsin, United States of America; Harvard University, UNITED STATES

## Abstract

EBV transforms B cells *in vitro* and causes human B-cell lymphomas including classical Hodgkin lymphoma (CHL), Burkitt lymphoma (BL) and diffuse large B-cell lymphoma (DLBCL). The EBV latency protein, EBNA2, transcriptionally activates the promoters of all latent viral protein-coding genes expressed in type III EBV latency and is essential for EBV’s ability to transform B cells *in vitro*. However, EBNA2 is not expressed in EBV-infected CHLs and BLs in humans. EBV-positive CHLs have type II latency and are largely driven by the EBV LMP1/LMP2A proteins, while EBV-positive BLs, which usually have type I latency are largely driven by c-Myc translocations, and only express the EBNA1 protein and viral non-coding RNAs. Approximately 15% of human BLs contain naturally occurring EBNA2-deleted viruses that support a form of viral latency known as Wp-restricted (expressing the EBNA-LP, EBNA3A/3B/3C, EBNA1 and BHRF1 proteins), but whether Wp-restricted latency and/or EBNA2-deleted EBV can induce lymphomas in humanized mice, or in the absence of c-Myc translocations, is unknown. Here we show that a naturally occurring EBNA2-deleted EBV strain (P3HR1) isolated from a human BL induces EBV-positive B-cell lymphomas in a subset of infected cord blood-humanized (CBH) mice. Furthermore, we find that P3HR1-infected lymphoma cells support two different viral latency types and phenotypes that are mutually exclusive: 1) Large (often multinucleated), CD30-positive, CD45-negative cells reminiscent of the Reed-Sternberg (RS) cells in CHL that express high levels of LMP1 but not EBNA-LP (consistent with type II viral latency); and 2) smaller monomorphic CD30-negative DLBCL-like cells that express EBNA-LP and EBNA3A but not LMP1 (consistent with Wp-restricted latency). These results reveal that EBNA2 is not absolutely required for EBV to form tumors in CBH mice and suggest that P3HR1 virus can be used to model EBV positive lymphomas with both Wp-restricted and type II latency *in vivo*.

## Author summary

The EBV protein, EBNA2, is required for the ability of EBV to transform B cells *in vitro*, but EBV-infected cells that express EBNA2 (and its downstream viral gene targets) are highly immunogenic and thus EBV-positive tumors in immunocompetent patients almost never express EBNA2. Currently, there are no model systems for studying how EBV causes B-cell lymphomas in the absence of EBNA2 expression in the context of the intact viral genome. Here we show that a naturally occurring EBNA2-deleted EBV strain (P3HR1) isolated from a human BL causes two different types of lymphomas in a cord blood-humanized mouse model. Some P3HR1-infected lymphoma cells phenotypically resemble the RS cell of CHL and express high levels of the viral LMP1 protein, a protein thought to drive EBV-positive human CHLs. The other P3HR1-induced lymphomas resemble DLBCLs and have a viral protein expression pattern consistent with Wp-restricted latency (EBNA-LP positive, LMP1 negative) similar to that occurring in human BLs infected with EBNA2-deleted EBV. These studies are the first to show that an EBNA2-deleted EBV can cause lymphomas in humanized mice with restricted forms of viral latency that resemble human CHLs and DLBCLs.

## Introduction

Epstein-Barr virus (EBV) is a human herpesvirus that contributes to a variety of different lymphomas in humans and transforms normal B cells *in vitro* into immortalized lymphoblastoid cell lines (LCLs). EBV-associated B-cell lymphomas include diffuse large B-cell lymphomas (DLBCLs), Burkitt lymphomas (BLs), classical Hodgkin lymphomas (CHLs), plasmablastic lymphomas, primary effusion lymphomas, primary CNS lymphomas, and a spectrum of post-transplant lymphoproliferative disorders including marginal zone lymphoma [1–5]. EBV can infect cells in either lytic or latent forms of infection. The lytic form of infection is required for production of infectious viral particles and horizontal transmission of the virus from cell to cell (and host to host), while the latent forms of infection allow the virus to persist long-term in memory B cells and evade the host immune response. However, there are several different gene expression patterns observed in latent EBV infection (commonly referred to as type I, type II and type III) that differ in regard to the number of viral proteins expressed, whether the virus can transform B cells *in vitr*o, and the ability of T cells to recognize and kill EBV-infected B cells *in vivo*.

B cells containing the most transforming, and immunogenic, form of EBV latency (type III) express all 9 EBV encoded latency proteins. Type III latency is the only EBV latency program that transforms B cells *in vitro*, and EBV-positive lymphomas with type III latency are largely (if not exclusively) driven by EBV-encoded oncogenes. The two major EBV transforming proteins, EBNA2 and LMP1, mimic constitutively active Notch and CD40 signaling, respectively, and are each essential for the ability of EBV to transform B cells *in vitro*. EBNA2 (which directly interacts with the cellular RBP-Jk (RBPJ) protein [6,7]) drives transcription of all the latent viral protein-coding genes during type III latency, and induces cellular proliferation by activating cellular genes such as c-Myc and Cyclin D [6,7]. However, since type III latency is also highly immunogenic, EBV-induced lymphomas with type III latency are largely confined to immunosuppressed hosts (such as transplant recipients and AIDS patients). In contrast, EBV-induced lymphomas occurring in more immunocompetent individuals, including BL, CHL and DLBCLs, usually have more stringent (and less immunogenic) forms of EBV latency, in which EBNA2 expression is turned off and fewer viral proteins are expressed [2,4]. Since stringent types of viral latency which have no EBNA2 expression are non-transforming *in vitro*, it has been difficult to model how EBV infection promotes lymphomas in the absence of EBNA2 expression.

EBV-positive tumors with type II or type I latency are responsible for the great majority of human deaths due to EBV-positive malignancies worldwide [8]. In EBV-infected B cells that have type II latency, EBNA2 expression is shut off, and the viral proteins EBNA1, LMP1 and LMP2A, along with the viral microRNAs and small nuclear RNAs (EBERs), are the only viral gene products expressed. LMP1 and LMP2A (which mimic the effects of constitutively active CD40 and B-cell receptor (BCR) signaling, respectively) are thought to be the major drivers of lymphomas with type II latency. While EBNA2 activates the LMP1/LMP2A promoters in cells with type III latency, in cells with type II latency, the LMP1/LMP2A promoters are activated via cellular proteins (including STAT 3/5 and NF-κB) [9–11]. Furthermore, EBNA1 is derived from an alternative viral Q promoter (Qp) rather than the EBNA2-driven viral C promoter (Cp) used to direct transcription of all EBNA genes during type III latency. While EBV-negative CHLs commonly have mutations that activate NF-κB, such mutations are much less frequent in EBV-positive CHLs, presumably since LMP1 expression activates NF-κB (reviewed in [12,13]). In addition, in EBV-infected human CHLs that have sterile BCR rearrangements, LMP2A expression is thought to substitute for authentic BCR signaling [14–16]. There are currently no *in vitro* or *in vivo* model systems for deriving stably EBV-transformed B cells that have type II latency in the context of the intact viral genome, although combined transgenic expression of both LMP1 and LMP2A in mouse germinal center cells induces massive plasmablast proliferation when T cells and NK cells are depleted [17].

All human EBV-positive BLs have either type I or Wp-restricted EBV latency. Type I latency (in which EBNA1 is the only protein expressed, along with virally-encoded miRNAs and EBERs) occurs when the two potential EBNA2 promoters (Cp and Wp) become methylated through poorly understood mechanisms, and EBNA1 expression continues using the Qp [6,7]. In Wp-restricted latency, which occurs in approximately 15% of human Burkitt lymphomas, the EBNA2 gene is deleted in a stereotypical manner that allows the latent W promoter (Wp) to drive expression of the EBNA1, EBNA-LP, EBNA3A/B/C and BHRF1 genes, resulting in a greater number of EBV proteins expressed relative to type I latency [18–22]. Both type I and Wp-restricted latency are completely unable to transform B cells *in vitro*.

Both EBV-positive and EBV-negative BLs are largely driven by c-Myc translocations. The major oncogenic role of EBV in BLs is thought to be the ability of stringent latent EBV infection to inhibit c-Myc-induced apoptosis [18–20,22,23]. Interestingly, BL cell lines with Wp-restricted latency are more dependent upon the continued presence of the EBV genome for growth and survival *in vitro* compared to BL cells line with type I latency [20], suggesting that viral proteins specifically expressed in cells with Wp-restricted latency contribute to Wp-restricted BL pathogenesis.

To date, EBV-positive human DLBCLs have been reported to contain a variety of different latency types, including type I, type II and type III viral latency. Interestingly, while DLBCLs with type III latency can occur in AIDS patients, the majority of AIDS-related DLBCLs have either type II or type I latency [5]. The precise roles(s) of different EBV latency programs in promoting human DLBCLs, and whether Wp-restricted latency ever occurs in human DLBCLs, has not been well studied.

The growth of CHLs and DLBCLs *in vivo* is strongly dependent upon the tumor microenvironment [24–30]. In fact, due to their extreme dependence on the *in vivo* microenvironment, most human lymphomas (particularly CHLs [31–33]) cannot be cultured *in vitro*, and those are not adequately reflect normal interactions between the tumor and the microenvironment. Furthermore, the only EBV-positive CHL cell line available, L591, has switched to type III latency [34], likely reflecting the inability of cells with type II latency to grow *in vitro* in the absence of the *in vivo* microenvironment and EBNA2. T cells are a particularly important component of the lymphoma microenvironment, especially in CHLs [31–33]. The malignant Reed-Sternberg (RS) cells in CHLs, which comprise only a small portion of the total tumor mass, depend upon growth factors provided by surrounding CD4 T cells (such as CD40L) to proliferate [31–33]. RS cells express factors (including galectin-1, CCL17 and CCL22) that recruit CD4 T cells to the tumor, and promote their differentiation into TH2 and Treg cells [35–38]. Of note, each of these RS-expressed factors is activated by LMP1 *in vitro* [39,40]. HLs are also often heavily infiltrated with collagen, and LMP1 activates expression of a collagen-stimulated receptor tyrosine kinase (DDR1) that may allow collagen in the tumor microenvironment to enhance survival of EBV-positive RS cells [41,42]. Thus, EBV-infected B cells with stringent latency types that are not transforming *in vitro* may receive growth and survival factors from CD4 T cells and/or collagen in the tumor microenvironment that allow these cells to induce lymphomas *in vivo*.

Humanized mice provide sophisticated models for understanding the complex interactions between EBV-infected B cells and the tumor microenvironment during the development of lymphomas with more stringent forms of viral latency. We have previously developed a novel cord blood-humanized (CBH) mouse model in which NOD/SCID/γC (NSG) mice are reconstituted with human cord blood cells that are infected with either wild-type or mutant EBV [43–45]. This model allows B cells and T cells to engraft into mouse splenic follicles and abdominal lymph nodes, and to interact in lymphomas. Importantly, the highly supportive microenvironment of the CBH model (combined with the less vigorous cytotoxic T cell response) allows some EBV mutants (including EBNA3C and LMP1 mutants) that are non-transforming *in vitro* to form lymphomas *in vivo* [43–45]. Using this model, we discovered that an LMP1-deleted EBV mutant causes activated DLBCLs *in vivo* because CD40L-expressing CD4 T cells in the tumor microenvironment can substitute for LMP1 [43] in lymphomas with type III latency that express EBNA2. In contrast, approximately 50 mice injected with uninfected cord blood have not developed B cell lymphomas in this same model. We also recently used the CBH model to show that type 2 EBV, although much less transforming than type 1 EBV *in vitro* due to reduced LMP1 expression [46]), is fully competent for inducing B-cell lymphomas *in vivo* [47].

In this manuscript, we have examined the *in vivo* phenotype of a naturally occurring EBNA2-deleted type 2 EBV strain, P3HR1 (originally isolated from a human Burkitt lymphoma), in cord blood-humanized mice. Surprisingly, although the P3HR1 virus is completely unable to transform normal human B cells *in vitro* [48], we find that P3HR1 infection in the CBH model results in EBV-infected B-cell lymphomas in a subset of mice. Furthermore, we show that some of the P3HR1 lymphomas resemble classical Hodgkin lymphomas and have type II latency, while other P3HR1 infected lymphomas resemble activated DLBCLs and support Wp-restricted latency. These results are the first to present an *in vivo* model in which EBV causes lymphomas in the absence of EBNA2 expression, and the first to demonstrate a system for generating Hodgkin-like lymphomas *in vivo* with type II latency.

## Results

### EBNA2-deleted P3HR1 virus induces B cell lymphomas in a subset of infected cord blood-humanized mice

**Fig 1:**
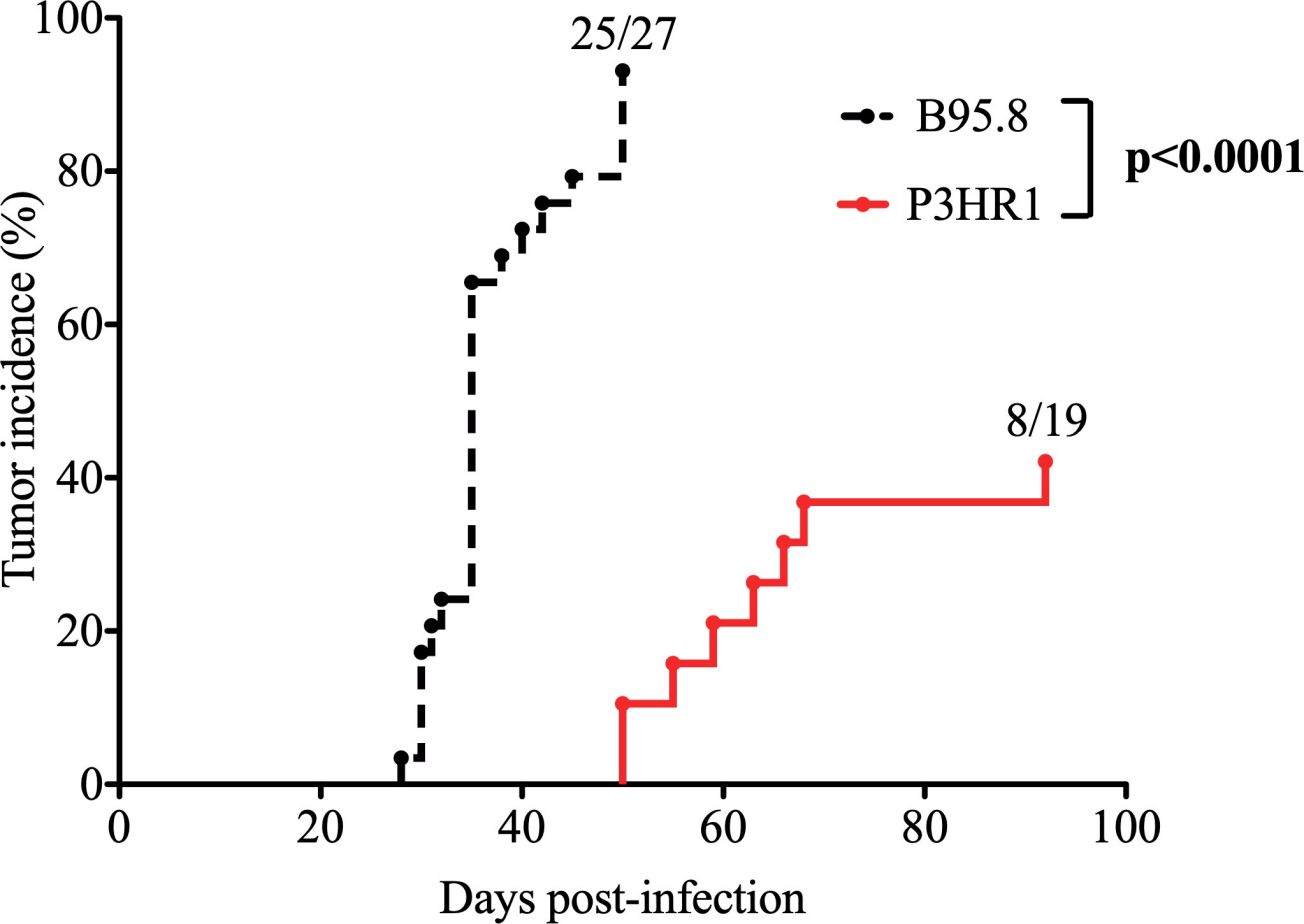
A subset of P3HR1 strain EBV-infected CBH mice develop tumors. Kaplan-Meier analysis was performed comparing tumor development over time (tumor incidence curve) in CBH mice infected with P3HR1 (EBNA2-deleted) virus versus B95.8 (WT, EBNA2-sufficient) virus. B95.8 data was previously published [44]. P3HR1 infected mice were infected with a 25- to 50-fold higher dose of virus compared to B95.8 infected mice as described in the methods. Log-Rank test was performed on tumor incidence curves.

To determine if loss of EBNA2 gene expression abrogates the ability of EBV to induce lymphomas in the cord blood-humanized (CBH) mouse model, we isolated virus from the P3HR1 Burkitt lymphoma (BL) line. P3HR1 virus is a type 2 EBV strain that contains a naturally occurring EBNA2 deletion and is completely unable to transform B cells *in vitro* [48]. Surprisingly, we found that approximately 40% of the P3HR1 virus infected CBH mice developed EBV-infected lymphomas at late time points (50–92 days after infection) (**Fig 1**). In comparison, we have previously reported that almost all CBH mice infected with B95.8 type 1 EBV develop lymphomas (usually 30 to 40 days after infection), and have never observed EBV-infected B-cell lymphomas in mice injected with cord blood alone [44]. These results indicate that EBNA2 is not absolutely required for the ability of EBV to induce lymphomas in the CBH model *in vivo*.

### Lymphomas induced by the P3HR1 virus in CBH mice have two different latency types (type II latency and Wp-restricted latency)

**Fig 2:**
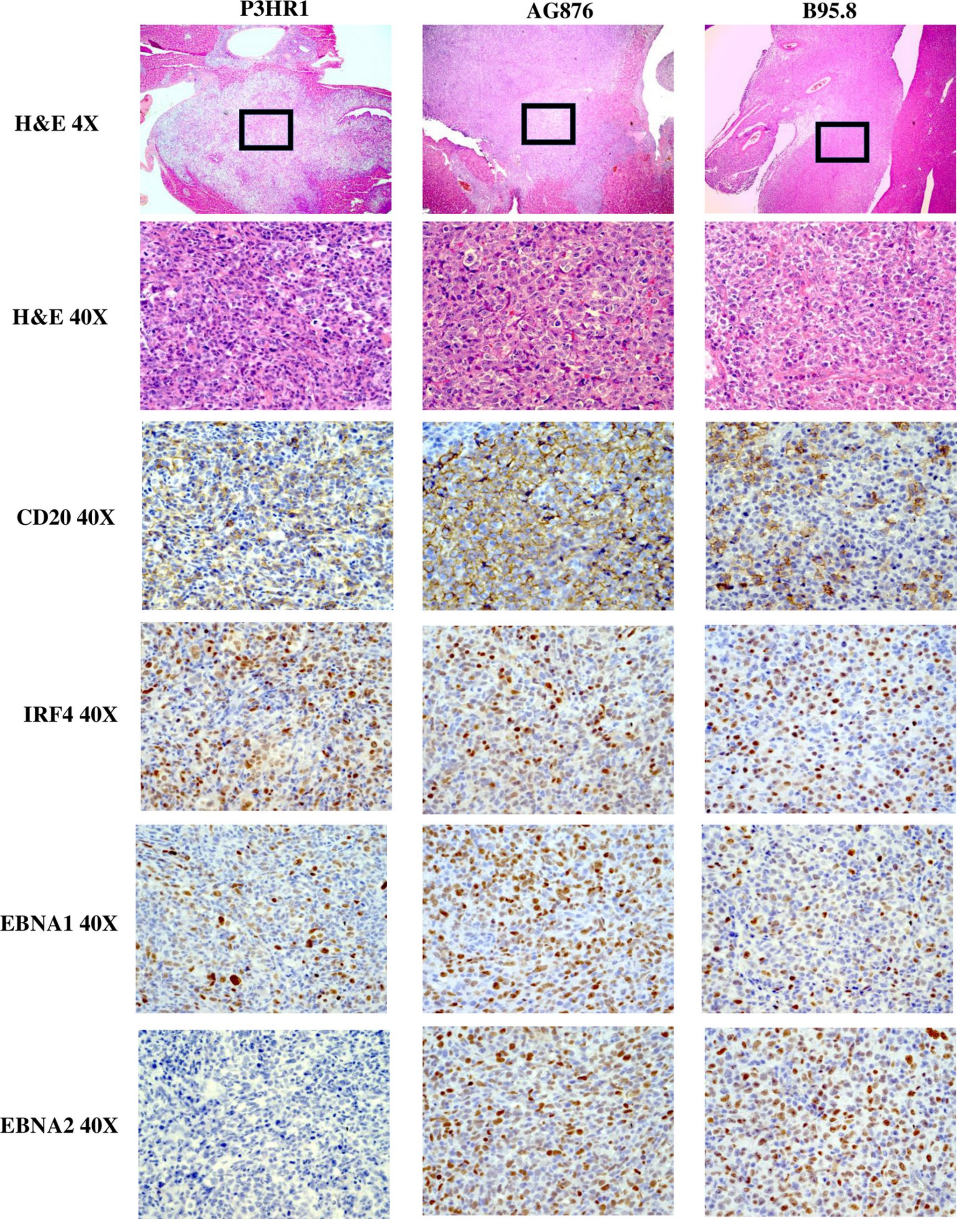
P3HR1 virus induces invasive B cell lymphomas. H&E staining was performed on P3HR1 virus, B95.8 virus or AG876 virus induced lymphomas (each invading the liver) as shown. In addition, IHC analysis was performed using antibodies against CD20 (B cell marker), IRF4 (marker of activated B cells), EBNA1 (EBV latency protein expressed in all EBV-infected cells) and EBNA2 (EBV latency protein expressed in cells with type III latency that is deleted in the P3HR1 virus). The P3HR1 tumor shown is from mouse #6 in **S1 Table**.

To examine the phenotypes of P3HR1 virus-induced lymphomas, formalin-fixed paraffin embedded (FFPE) tissues from animals bearing tumors were subjected to hematoxylin and eosin (H&E) stain. Tumors obtained from CBH mice infected with either B95.8 EBV (an EBNA2-expressing type 1 EBV strain) or AG876 EBV (an EBNA2-expressing type 2 EBV strain) were also included in this analysis. P3HR1 virus induced highly aggressive B cell lymphomas in CBH mice that stained positive for the B-cell marker CD20 and IRF4 (a marker for activated, non-germinal center B cells also known as MUM-1) (**Fig 2**) and invaded multiple organs including pancreas, liver, biliary tract, kidney, mesentery, abdominal lymph node, spleen and (rarely) bone (**Table 1** and **S1 Fig)**.

**Table 1:** Summary of P3HR1 tumor morphologies.

Mouse #	Tumor morphology
1	DLBCL with foci of CHL like process with syncytial RS-like cells in liver and pancreas
2	DLBCL with foci of CHL like process with syncytial RS-like cells in kidney
3	DLBCL with intermingled larger, RS-like cells in liver and bile duct
4	CHL like tumor with invasion of vertebral body
5	DLBCL involving liver, kidney, pancreas, and spleen with intermingled RS-like cells in spleen
6	DLBCL with intermingled larger, RS-like cells in spleen, abdominal wall, serosa, kidney
7	DLBCL with intermingled larger, RS-like cells in pancreas and mesentery
8	DLBCL in pancreas and more CHL like lymphoma in spleen and liver.

The tumor morphology observed in 8 different P3HR1 infected mice is described. DLBCL: diffuse large B cell lymphomas. CHL: Classical Hodgkin lymphoma. RS: Reed-Sternberg cell

To compare EBV protein expression in lymphomas infected with each type of virus, FFPE tissues isolated from CBH mice infected with P3HR1, AG876 or B95.8 EBV strains were initially stained with antibodies against EBNA1 (a latency protein expressed in cells with any latency type) and EBNA2 (a latency protein expressed only in cells with type III latency). As expected, both B95.8 and AG876 virus-induced lymphomas (which have previously been shown to have type III latency in CBH mice)[43–45,47], express both EBNA1 and EBNA2 (**Fig 2**). In contrast, tumors derived from the EBNA2-deleted P3HR1 virus express EBNA1 but not EBNA2 (**Fig 2**). The lack of EBNA2 expression in the P3HR1 virus-infected lymphomas confirms that these tumors are not derived from contaminating EBV in the cord blood. In addition, as shown in **S2 Fig,** we found that expression of the EBV latency protein, EBNA1, is confined to CD20 positive cells (B cells) and is not observed in CD3 positive cells (T cells), confirming that P3HR1 virus induced lymphomas are B-cell lymphomas in this CBH mouse model.

**Fig 3:**
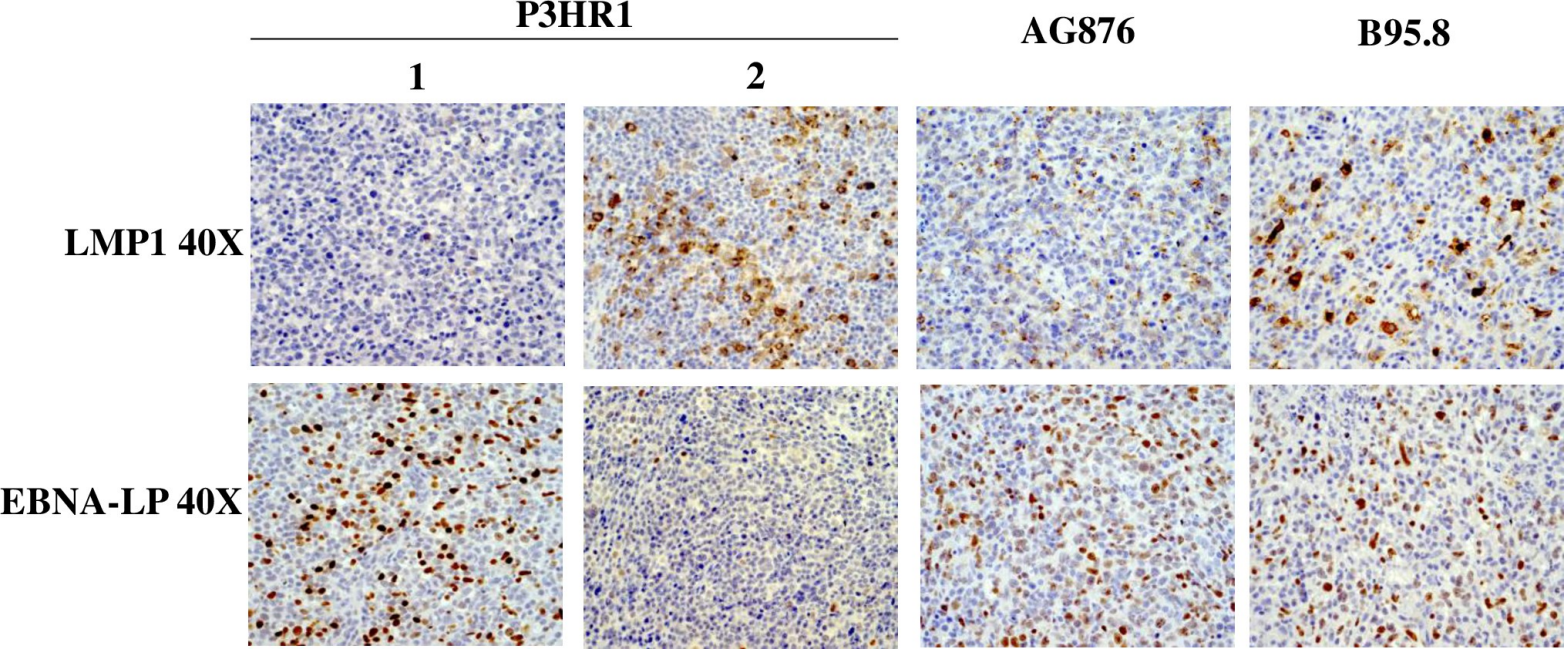
P3HR1 infected lymphomas contain foci with Wp-restricted latency as well as foci with type II latency. IHC analysis using antibodies against EBNA-LP (expressed in type III latency and Wp-restricted latency) or LMP1 (expressed in type II and type III latency) was performed as indicated in tumors infected with either the P3HR1 virus, B95.8 virus or AG876 virus. P3HR1 “1” is from mouse #3 and P3HR1 “2” is from mouse #6 in **S1 Table**.

We next examined the level of EBNA-LP expression (a latency protein expressed in cells with type III or Wp-restricted latency, but not type II or type I latency) and LMP1 expression (a latency protein expressed in type III and type II latency but not Wp-restricted latency) in each tumor type. EBNA-LP, a transcriptional co-activator, is the first viral protein (along with EBNA2) to be expressed in newly infected B cells and can be derived from either the EBV Wp (the initial viral promoter used to express EBNA transcripts in newly infected B cells) or the viral Cp (which is used exclusively to drive expression of all EBNA genes in cells with type III latency). As expected, lymphomas infected with the B95.8 virus express both EBNA-LP and LMP1 in a significant number of tumor cells (**Fig 3**). Lymphomas infected with type 2 EBV AG876 virus, as we recently reported [47], express less LMP1 compared to type 1 EBV B95.8 infected cells but have similar levels of EBNA-LP (**Fig 3**).

Surprisingly, however, whereas some areas of P3HR1 virus infected lymphoma cells express high levels of EBNA-LP (for example, P3HR1 “1” in **Fig 3**), other P3HR1 infected tumor foci have little or no EBNA-LP expression (for example, P3HR1 “2” in **Fig 3**). Furthermore, we found that areas of P3HR1 virus infected tumors that express high levels of EBNA-LP have very low levels of LMP1 (P3HR1 “1”), consistent with Wp-restricted latency, while areas that express low levels of EBNA-LP have high levels of LMP1 (P3HR1 “2”) (**Fig 3**), suggesting type II latency. All P3HR1 infected animals contained some tumor foci suggestive of Wp-restricted latency, and other tumor foci suggestive of type II latency.

### Wp-restricted and type II Latency are mutually exclusive in P3HR1 virus-infected lymphoma cells

**Fig 4:**
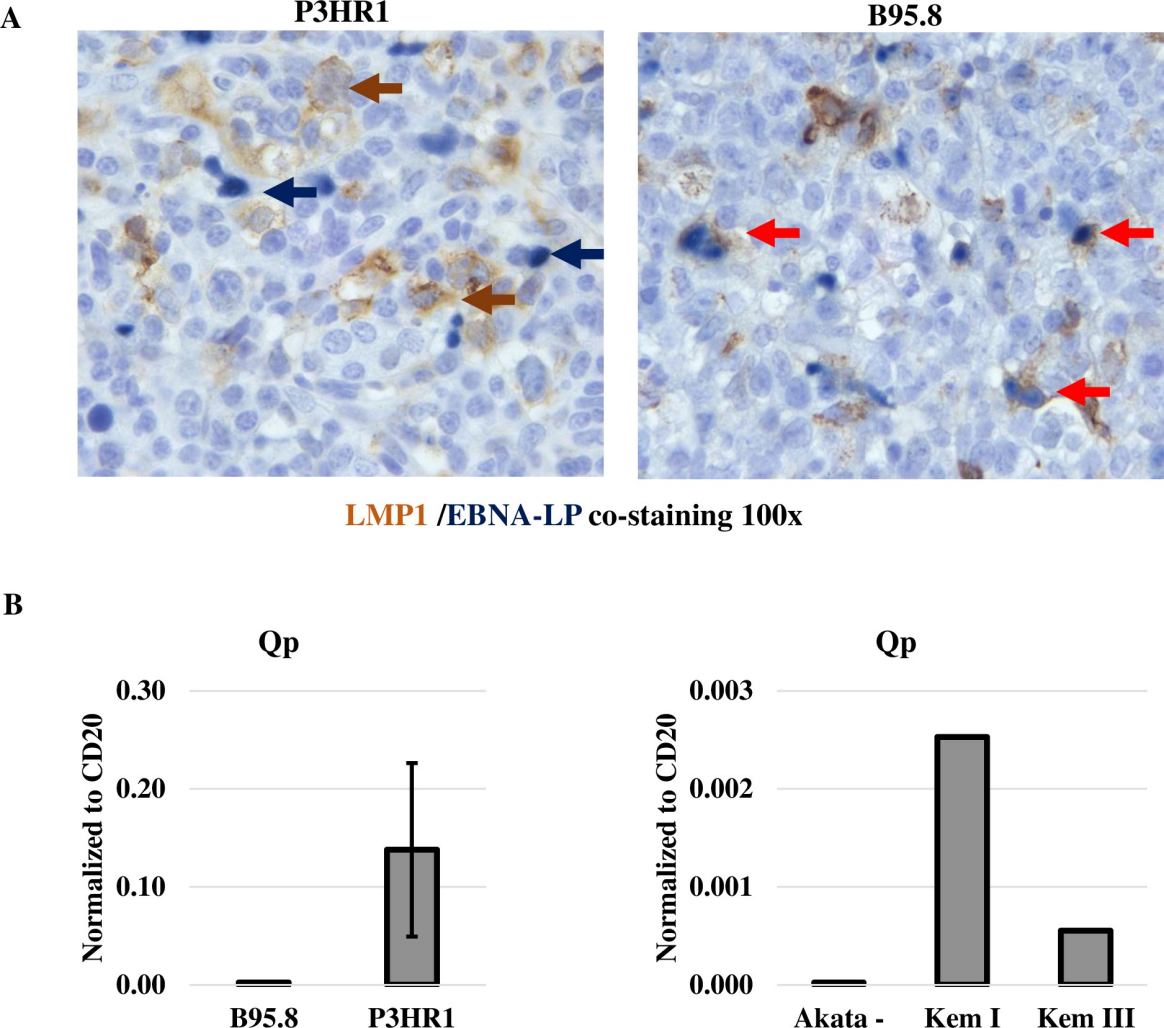
LMP1 expression and EBNA-LP expression are mutually exclusive in P3HR1 infected lymphomas in CBH mice, and the type 1/II latency EBNA1 Qp promoter is used in P3HR1 tumors. **A.** IHC co-staining was performed using an antibody against EBNA-LP (dark blue staining) and an antibody against LMP1 (brown staining) in lymphomas infected with P3HR1 or B95.8 viruses. Examples of cells that express only EBNA-LP are indicated by blue arrows, and examples of cells expressing only LMP1 by brown arrows. Co-staining cells are indicated by red arrows. The P3HR1 tumor shown in from mouse #2 in **S1 Table**. **B.** qPCR analysis of RNA isolated from P3HR1 and B95.8 EBV-induced lymphomas was performed using primers that recognize EBNA1 derived from the Qp promoter (type I/type II latency specific) (left panel); qPCR analysis of RNA isolated from BL control cell lines (Kem I and Kem III) with type I and type III latency, respectively, and EBV-negative Akata BL cells served as positive and negative controls in these assays (right panel).

Cells containing type III EBV latency are thought to simultaneously express EBNA-LP and LMP1 (with the promoters of both viral transcripts being activated by EBNA2). However, given that the EBNA-LP and LMP1 single stain studies in the P3HR1 virus-infected animals suggest that EBNA-LP and LMP1 are often expressed in geographically different regions of the tumors, we next performed EBNA-LP/LMP1 co-staining studies to determine if EBNA-LP and LMP1 are expressed in the same cells, or separate cells, in P3HR1 virus-infected lymphomas. As shown in **Fig 4A**, EBNA-LP and LMP1 co-staining cells can be detected in B95.8 virus infected lymphomas with type III latency, as expected. However, in P3HR1-infected lymphomas, cells stained positive for either EBNA-LP or LMP1, but not both. These results suggest that while a subset of P3HR1 infected tumor cells have Wp-restricted viral latency (similar to what is observed in human Burkitt lymphoma tumors infected with EBNA2-deleted EBV strains), other P3HR1 infected lymphoma cells appear to be in type II latency, as they express LMP1 but not EBNA-LP.

Since cells with type II and type I EBV latency use a distinct EBV promoter (Qp) to drive EBNA1 transcription (in contrast to the Cp and Wp promoters used in type III and Wp-restricted latency, respectively), we next performed RT-PCR on RNA isolated from P3HR1 infected tumor tissue versus B95.8 virus infected tumor tissue using PCR primers previously shown to specifically detect EBNA1 transcripts derived from the Qp [49]. Burkitt lymphoma control cell lines (Kem I and Kem III) with type I and type III latency, respectively, and EBV-negative Akata BL cells served as positive and negative controls in these assays (**Fig 4B**). As shown in **Fig 4B**, Qp-derived transcripts were observed in P3HR1 virus infected tumors, but not the B95.8 virus infected tumors. Primers specific for the Cp and Wp promoters, although giving the expected results in control BL cell lines, appeared to non-specifically recognize mouse RNAs since they resulted in very high values of transcript in all tumor types (and hence could not be used). Together, these results confirm that some of the P3HR1-infected tumor cells use the Qp EBNA1 promoter and have type II viral latency.

### LMP1-positive P3HR1 tumor cells express CD30 and have Reed-Sternberg (RS) like characteristics

**Fig 5:**
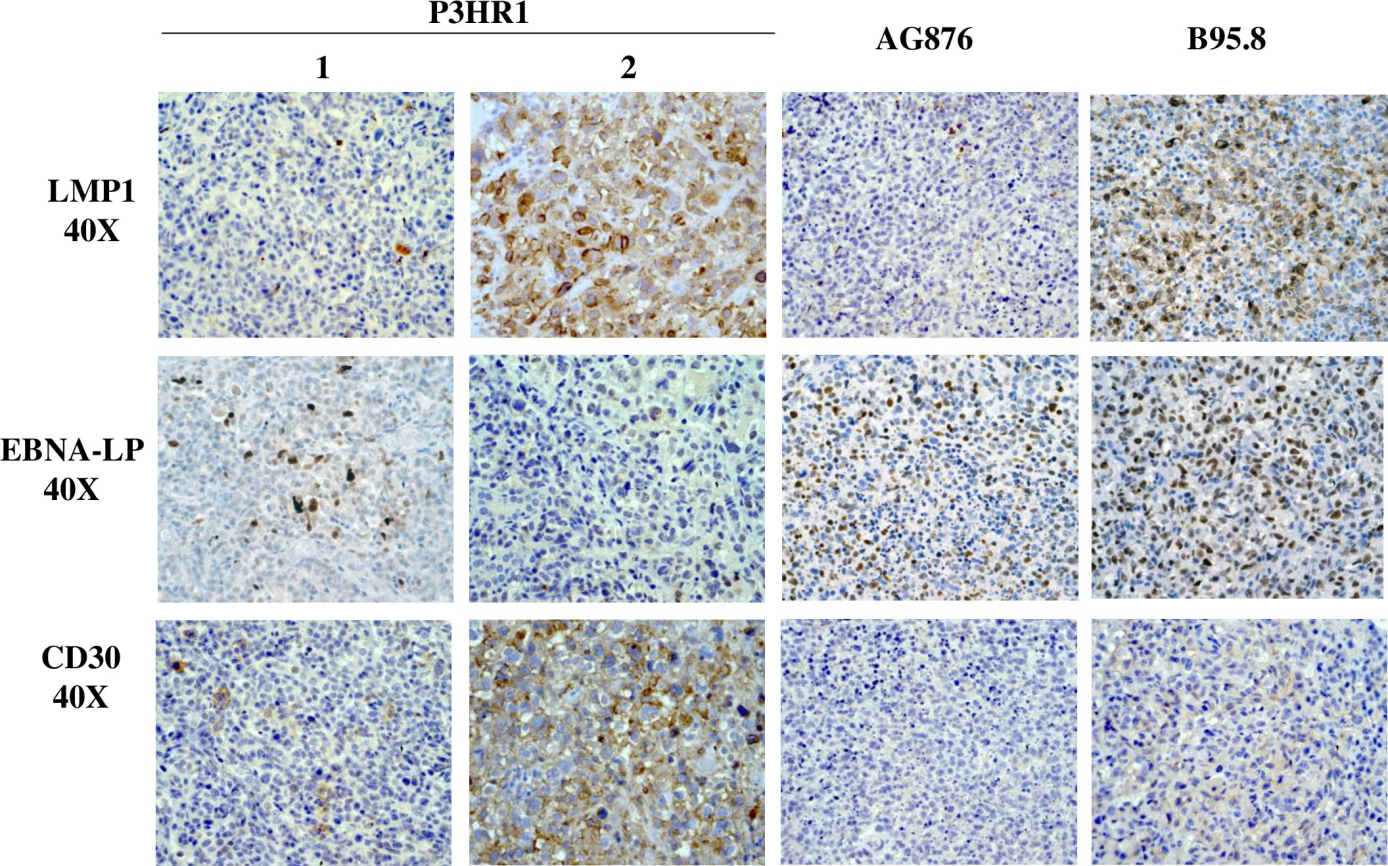
P3HR1-infected lymphoma cells with high level LMP1 expression also have high level CD30. IHC analysis of P3HR1 infected, AG876 infected or B95.8 virus infected lymphomas were performed using antibodies that detect LMP1, EBNA-LP or CD30 as indicated. P3HR1 “1” and P3HR1 “2” are derived from geographically separated tumor foci in the kidney of mouse #2 in **S1 Table**.

Since EBV-positive human classical Hodgkin lymphomas have type II EBV latency and express a very high level of CD30, we asked if the LMP1-high/EBNA-LP-low P3HR1 lymphoma cells express more CD30, or have a morphology more characteristic of Hodgkin lymphoma-like cells, in comparison to the EBNA-LP-high/LMP1-low P3HR1 virus infected lymphoma cells. As shown in **Fig 5,** P3HR1 tumor foci characterized by LMP1-high/ EBNA-LP-low cells (such as P3HR1 “2”) express much more CD30 in comparison to either the EBNA-LP-high/LMP1-low P3HR1 virus infected cells (such as P3HR1 “1”), or in comparison to either B95.8 or AG876 virus infected lymphoma cells with type III latency.

**Fig 6:**
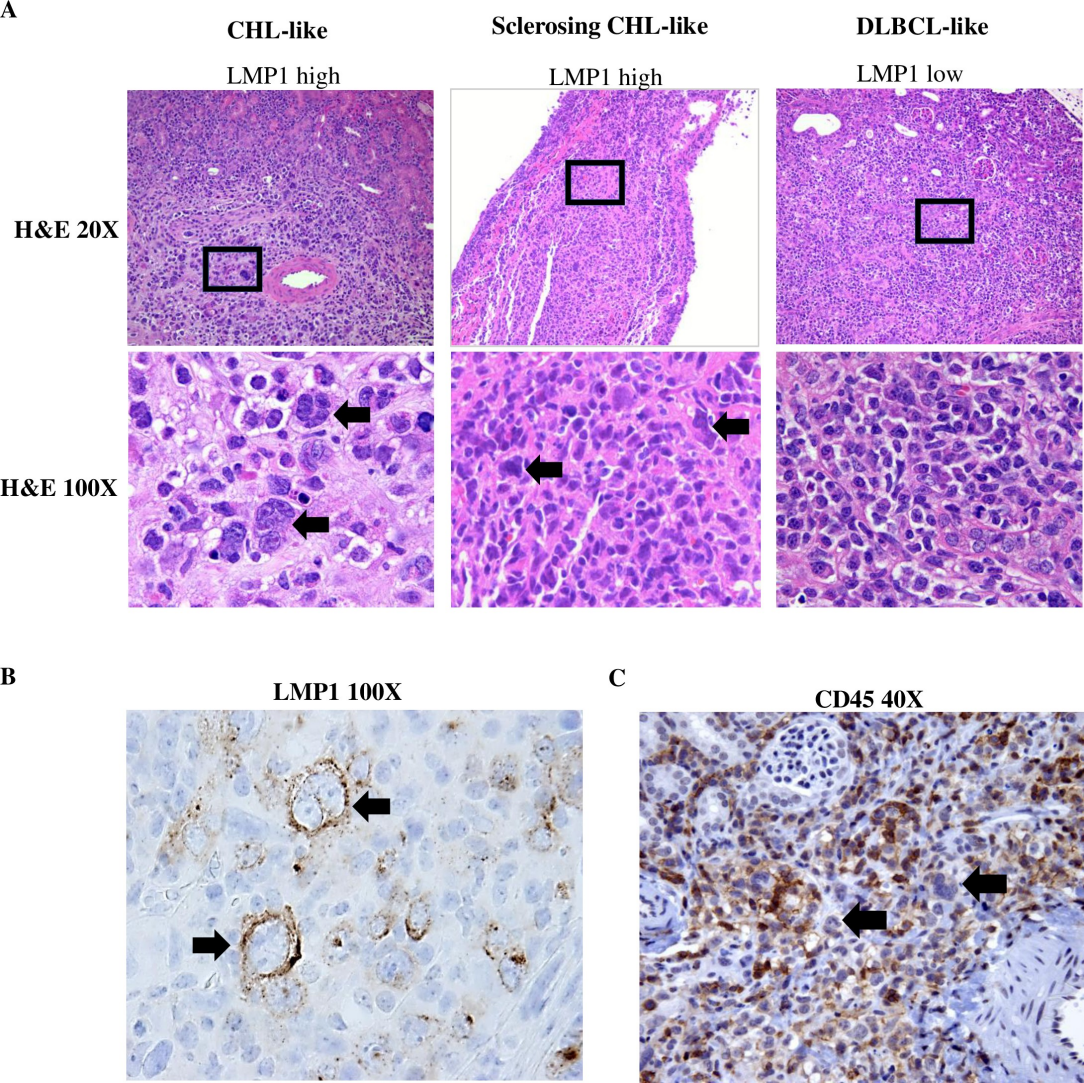
LMP1-high/EBNA-LP-low cells in P3HR1 virus infected lymphomas resemble RS cells of CHLs. **A.** H & E staining was performed on P3HR1 virus infected lymphomas expressing high level LMP1/low level EBNA-LP (left panel: mouse #2; middle panel: mouse #7 in **S1 Table**), or low level LMP1/high level EBNA-LP (right panel: mouse #2 in **S1 Table**) as indicated at low power (20X) or high power (100X). **B.** LMP1 staining was also performed to show that RS-like cells in the LMP1 high/EBNA-LP-low tumor foci express LMP1 (mouse #6 in **S1 Table)**. **C**. CD45 staining of LMP1-high/EBNA-LP-low tumor regions shows loss of CD45 expression on large RS cells (black arrows) but continued CD45 expression on smaller cells (mouse #2 in **S1 Table)**.

Furthermore, as shown in **Fig 6A and S3 Fig**, LMP1-high/EBNA-LP-low tumor foci are composed of larger cells suggestive of a Hodgkin-like phenotype (including multinucleated cells with prominent, eosinophilic nucleoli, reminiscent of RS). Lesions containing RS cells in some cases showed evidence of sclerosis similar to that seen in nodular sclerosing CHL. In contrast to the LMP1-high/EBNA-LP-low cells, the EBNA-LP-high/LMP1-low tumor foci are composed of smaller monomorphic cells more consistent with a DLBCL-like phenotype. The RS-like cells in the LMP1-high/EBNA-LP-low regions of P3HR1 tumors stain positive for LMP1 (**Fig 6B**), confirming that they are EBV-infected. Furthermore, the RS-like cells observed in LMP1-high/EBNA-LP-low regions of P3HR1 tumors often lose expression of the cellular CD45 marker (**Fig 6C)**, as is commonly observed in human RS cells of HLs. CD20 expression was variably lost in the RS cells (**S4 Fig**).

### P3HR1 lymphomas express a mixture of Wp- and type II- associated latency proteins, as well as a high level of the BZLF1 lytic protein

**Fig 7:**
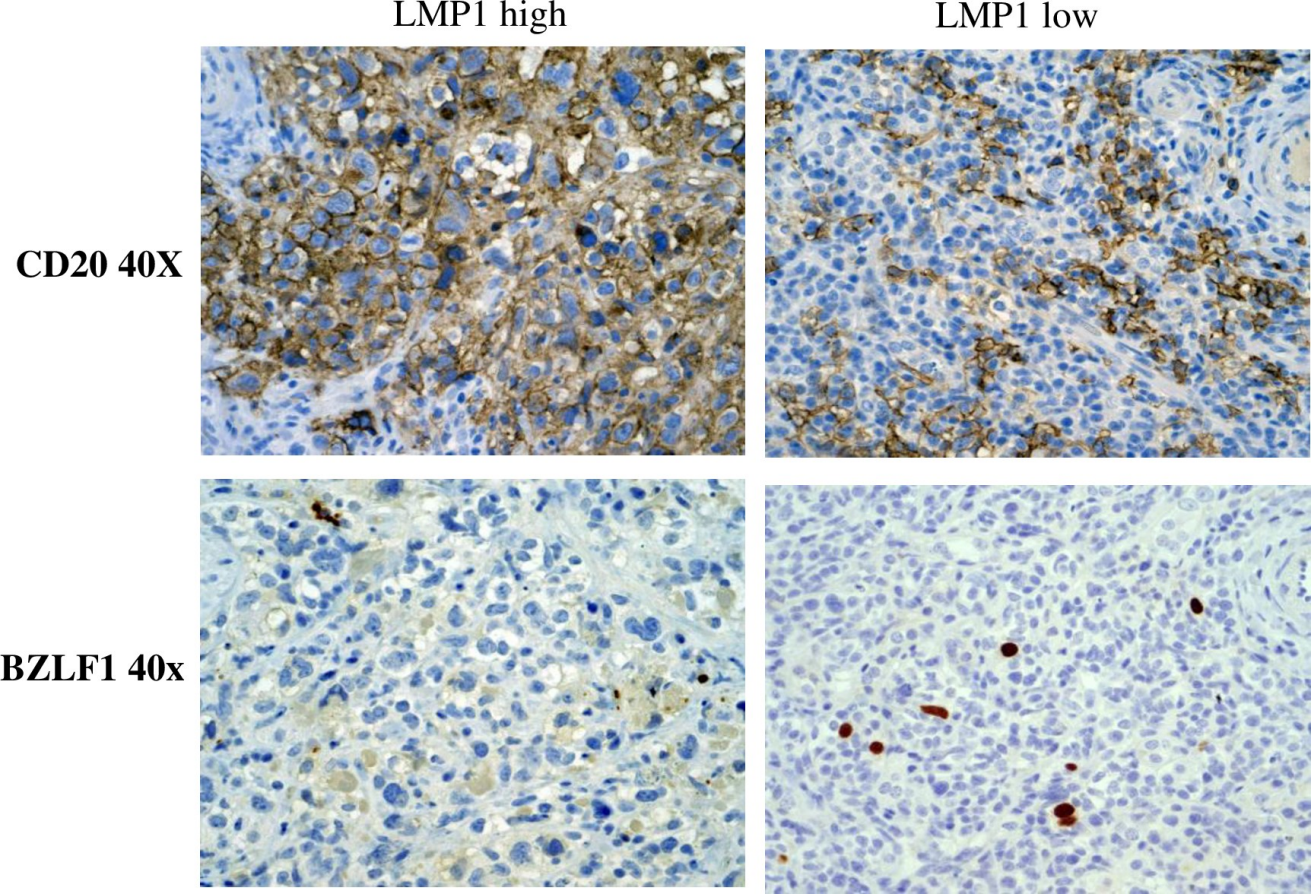
P3HR1-infected lymphoma cells express high level of the BZLF1 lytic viral protein in EBNA-LP-high/LMP1-low regions of tumors. IHC analysis of P3HR1 infected lymphomas was performed using antibodies that detect BZLF1 (lytic EBV protein) and CD20 (B cell marker). Both P3HR1 sections were derived from different tumor foci in the same kidney of mouse #2 in **S1 Table**.

**Fig 8:**
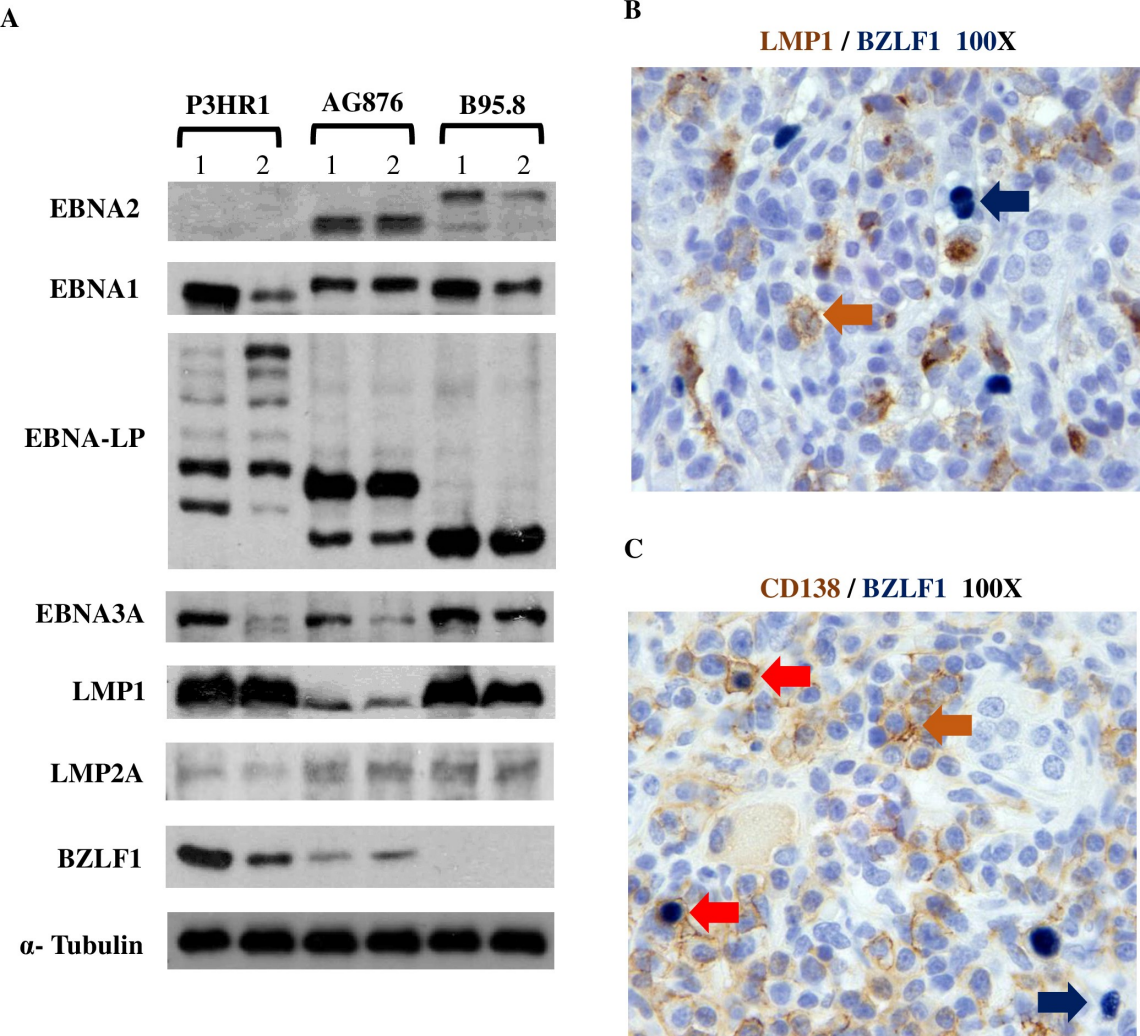
P3HR1 infected lymphomas express both Wp-restricted and type II viral latency proteins and the immediate-early lytic protein BZLF1. **A.** Immunoblot analysis of proteins isolated from P3HR1 infected, AG876 infected or B95.8 virus infected lymphomas was performed using antibodies against EBNA1, EBNA2, EBNA3A (EBV latency protein expressed in type III and Wp-restricted latency), LMP1 (EBV latency protein expressed in type II and III latency), LMP2A (EBV latency protein expressed in type II and type III latency), lytic protein BZLF1 or alpha tubulin (loading control). P3HR1 “1” protein is isolated from mouse #1 and P3HR1 “2” protein isolated from mouse #2 in **S1 Table**. EBNA-LP protein as expected can be expressed in multiple different sizes, and EBNA2 protein as expected is larger in type 1 EBV B95.8 infected tumors compared to type 2 EBV AG876 infected tumors. **B**. IHC co-staining studies were performed using antibodies that detect LMP1 or BZLF1 in a P3HR1 infected tumor (mouse #2 in **S1 Table**). No co-staining was observed. Blue arrow shows an example of an EBNA-LP staining cell, and brown arrow shows an example of an LMP1 positive cell. **C**. IHC co-staining studies were performed on a P3HR1 infected tumor using antibodies that detect BZLF1 or CD138. (mouse #2 in **S1 Table).** Blue arrow shows an example of a BZLF1 only staining cell, brown arrow shows an example of a CD138 only staining cell, and red arrows show examples of BZLF1/CD138 co-staining cells.

We also compared the level of the lytic immediate-early viral protein, BZLF1, in P3HR1 tumor foci that were LMP1-high/EBNA-LP-low versus EBNA-LP-high /LMP1-low using IHC analysis **(Fig 7).** We found that BZLF1-positive cells are much more common in the EBNA-LP-high/LMP1-low tumor foci compared to the LMP1-high/EBNA-LP-low foci. To more quantitatively compare the levels of different latent and lytic EBV proteins in lymphomas infected with the P3HR1, B95.8 or AG876 viruses, we isolated protein extracts from lymphomas containing each type of virus and performed immunoblots to quantitate EBNA2, EBNA-LP, EBNA3A, LMP1 and BZLF1 protein expression; we did not find any commercially available antibody that can detect type 2 EBV EBNA3C on immunoblot (or by IHC). As shown in **Fig 8A**, as expected P3HR1 virus infected tumors do not express EBNA2. However, both EBNA-LP and EBNA3A are clearly detected in the P3HR1-virus infected lymphomas, confirming that at least a subset of the P3HR1-infected tumor cells has Wp-restricted latency (since EBNA3A and EBNA-LP are not expressed in cells with type II latency). Although we did not detect BHRF1 expression, this result may reflect poor antibody staining, since we could also not detect BHRF1 expression in the Wp-restricted P3HR1 BL cell line (previously reported to express BHRF1).

Similar to our recent report comparing the phenotypes of type 1 versus type 2 EBV infected lymphomas in CBH mice [47], we found that type 1 B95.8 infected lymphomas express much more LMP1 than type 2 AG876 infected lymphomas, while AG876 infected lymphomas express much more of the BZLF1 lytic viral protein than B95.8 infected lymphomas (**Fig 8A**). In contrast, lymphomas induced by P3HR1 (although it is a type 2 EBV strain) express much more LMP1 compared to the AG876 type 2 control lymphomas, consistent with the IHC results in **Fig 3** and confirming that some P3HR1 virus infected cells have type II latency. LMP2A (expressed in type II and type III latency) is expressed at similar levels in lymphomas infected with the P3HR1, AG876 and B95.8 viruses (**Fig 8A**).

BZLF1 expression is similarly high in both P3HR1 and AG876 lymphoma cells (**Fig 8A**), suggesting that the lytic phenotype of P3HR1 tumors is likely a type specific trait rather than due to loss of EBNA2 expression per se. Co-staining of P3HR1 tumor cells with LMP1 and BZLF1 antibodies confirmed that BZLF1 and LMP1 expression are not observed in the same cells (**Fig 8B**). Given that plasma cell differentiation is associated with lytic EBV reactivation, we also performed CD138 and BZLF1 co-staining in EBNA-LP-high/LMP1-low areas of P3HR1 tumors. We found that some BZLF1 positive cells do co-express CD138 (**Fig 8C**), but others do not.

### RNA-seq analysis confirms high level lytic EBV gene expression in P3HR1 infected lymphomas and oligoclonality of tumors

**Fig 9:**
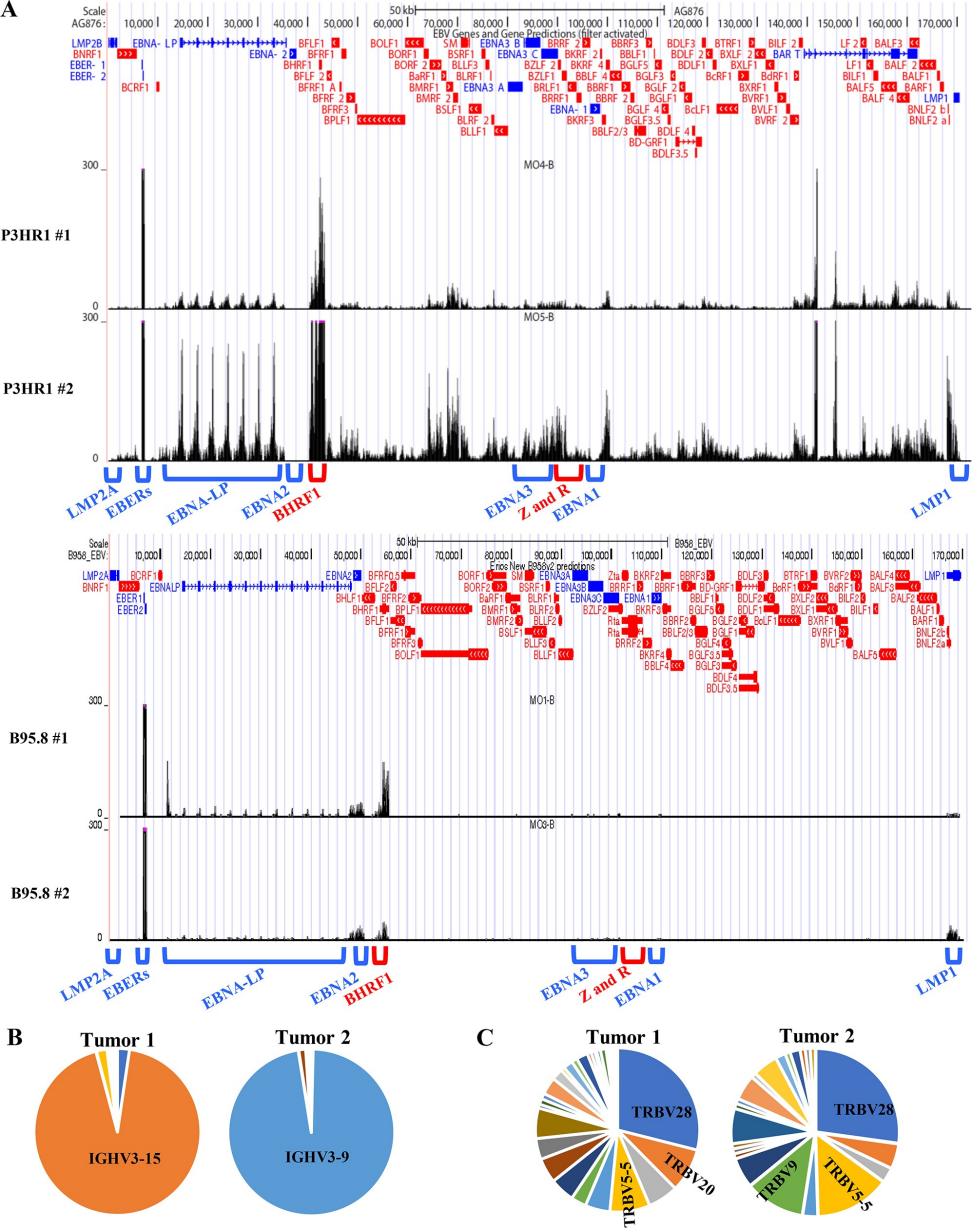
P3HR1 virus-infected tumors express a complex mix of latent and lytic genes and are oligoclonal. **A.** RNA-seq reads were mapped to the EBV genomes of 2 different tumors infected with P3HR1 virus (top panel), or two different tumors infected with B95.8 virus (middle panel). The locations of the Cp and the EBER, EBNA-LP, EBNA2, BHRF1, EBNA3, BZLF1 (Z), BRLF1 (R), EBNA1, LMP1 and LMP2A transcripts are indicated above the EBV genome map. P3HR1 “1” tumor is derived from mouse #6, and P3HR1 “2” tumor derived from mouse #7 in **S1 Table**. **B.** RNA-seq analysis was performed using RNA isolated from 2 different P3HR1 infected tumors shown in top panel in A. The relative frequency of the IGH transcripts containing various different IGHV genes is shown for each tumor. **C**. The frequency of the T cell receptor TRBV gene reads in the RNA-seq analysis of each P3HR1 tumor was determined by comparing the number of reads from each individual TRBV gene to the total number of TRBV gene reads.

RNA-seq analysis was performed on tumor tissues collected from two P3HR1-virus infected CBH mice and compared to previous results [44] obtained from tumor tissues collected from two B95.8 virus infected CBH mice. Consistent with the IHC and immunoblot analyses, analysis of viral transcripts in both P3HR1 virus infected-tumors suggests a high level of lytic viral gene transcription (**Fig 9A**). Both EBNA-LP, BHRF1 and LMP1 expression are clearly observed in P3HR1 tumor “2” (suggesting that the RNA isolated from this tumor contains a mixture of Wp-restricted latency cells as well as cells with type II latency); relatively less LMP1 transcript is expressed in the second P3HR1 tumor, although EBNA-LP and BHRF1 transcripts are easily detected. Of note, both P3HR1 tumors have much more EBNA-LP, BHRF1 and lytic gene expression in comparison to the two B95.8 virus infected tumors.

Interestingly, analysis of the BCR sequences (derived from RNA-seq data) of both P3HR1 virus infected lymphomas revealed that both tumors are largely monoclonal (**Fig 9B)**, indicating that they are derived from an expansion of a limited number of EBV-infected B cells [44]. In contrast, analysis of RNA-seq derived TCR sequences revealed a polycolonal T cell infiltrate (**Fig 9C**). Since at least the P3HR1 “2” tumor tissue used for RNAseq analysis contains cells with both LMP1-high/EBNA-LP-low expression, as well as cells with LMP1-low/EBNA-LP-high expression, the finding that the tumor is monoclonal suggests that cells with each of the two different forms of viral latency were originally derived from the same EBV-infected cell.

### Numerous cellular genes are differentially regulated in lymphomas infected with P3HR1 EBV versus B95.8 EBV, including cellular genes dysregulated in CHLs

**Fig 10:**
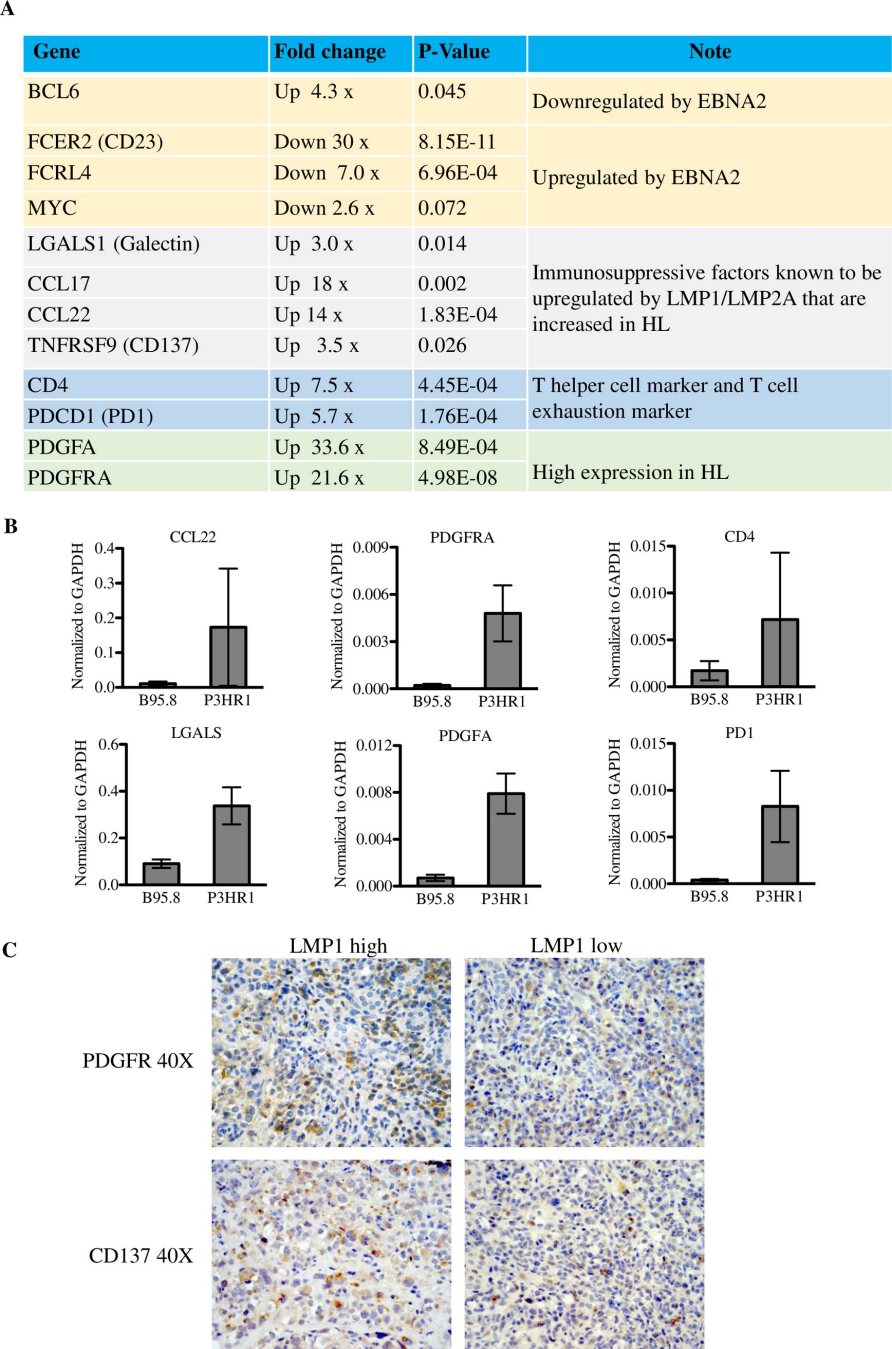
P3HR1 infected lymphomas express cellular genes upregulated in RS cells of Hodgkin lymphomas. RNA was isolated from tumors infected with B95.8 or P3HR1 virus-induced lymphomas, and RNA-seq performed. Mouse cell transcripts were removed from further analysis, and the levels of human genes in each tumor type was compared as described in the methods. **A.** The relative levels of cellular gene transcripts in P3HR1-infected tumors, versus B95.8-infected tumors, are shown for known EBNA2 target genes (yellow shading), immunosuppressive factors which are highly expressed in RS cells and are known LMP1 targets (gray shading), markers for helper T cells and T cell exhaustion (blue shading), and markers for enhanced PDGF signaling (which is often increased in HL tumors)(green shading). The fold-change in cellular gene expression in P3HR1-infected tumors versus B95.8-infected tumors is indicated, as well as the p-value for each difference. **B.** qPCR was performed using cDNA isolated from two different P3HR1 virus-induced lymphomas versus two different B95.8 virus-infected lymphomas, using human specific primers to amplify genes. Results were normalized to the level of GAPDH transcript. Standard error is shown. **C.** IHC analysis using antibodies against PDGFRA or CD137 was performed in areas of tumors with low or high level LMP1 expression. Both P3HR1 tumor sections shown are derived from mouse #2 (**S1 Table**).

RNA-seq analysis was further employed to compare the cellular gene expression in EBV-induced lymphomas in CBH mice infected with EBNA2 expressing B95.8 virus versus EBNA2-deleted P3HR1 virus. RNA-seq reads were aligned to the mouse and human genomes and mouse genomes were removed from the analysis. Numerous cellular genes were found to be differentially regulated in lymphomas infected with B95.8 virus versus P3HR1 virus (**S5 Fig**). Importantly, a number of immunosuppressive factors that are secreted at high levels by the RS cells in human CHLs, and which play key roles in recruiting CD4 T cells and promoting their differentiation into TH2 and Treg cells (including LGALS1 (galectin-1), CCL17 (TARC) and CCL22), are expressed at higher levels in P3HR1 tumors compared to B95.8 tumors (**Fig 10A**). Furthermore, LMP1 alone has previously been shown to induce CCL17 and CCL22 expression *in vitro* and the combination of LMP1 and LMP2A together induces galectin-1 expression [39,40]. Consistent with the ability of the CCL17 chemokine to induce chemotaxis of CCR4-expressing CD4 cells, P3HR1 tumors have increased expression of the CD4 gene (but not the CD8A gene), and (similar to CHLs) there is also high level of expression of T cell inhibitory factor PD1 in P3HR1 tumors, suggesting that the infiltrating CD4 T cells are exhausted (**Fig 10A**).

In addition, similar to what has been observed in human CHLs [50,51], both the PDGFA ligand and PDGFRA receptor genes are expressed at higher levels in P3HR1 HL-like tumors than B95.8 virus-induced tumors **(Fig 10A)**. Although EBV infection has not been reported to induce PDGFRA signaling in B cells, both PDGFα and PDGFRA are elevated in EBV-positive T/NK cell lymphomas with type II latency, and this pathway is important for their growth [52].

qPCR performed on cDNA isolated from WT B95.8 versus P3HR1 infected tumors confirmed that CCL22, LGALS1, PDGFRA, PDGFA, PDCD1 (PD1) and CD4 are expressed at higher levels in P3HR1 infected versus B95.8 EBV infected lymphomas (**Fig 10B)**. IHC analysis also revealed high levels of PDGFR1 expression in P3HR1 tumors in both the LMP1 high and LMP1 low regions of the tumors (**Fig 10C)**.

Another gene upregulated in P3HR1 lymphomas is TNFRSF9 (4-1BB; CD137) (**Fig 10A**). CD137, which is expressed at high levels in CHL RS cells [53], and is also a known target of LMP1 [54], has been suggested to protect RS cells from T cell mediated killing by sequestering the T cell receptor activating ligand, CD137L [53]. IHC analysis confirmed that CD137 is expressed at high levels especially in the LMP1 high regions of tumors (**Fig 10C**).

### C-Myc expression is variable in P3HR1 infected tumors

Although RNA-seq studies (**Fig 10A**) confirmed that P3HR1 tumors have the expected differences in three different known EBNA2 target genes, BCL6, FCER2 (CD23) and FCRL4 [55–58], the EBNA2 target gene transcript, c-Myc, is not significantly decreased although there is a trend toward decreased expression in the two P3HR1 tumors examined (**Fig 10A**). Protein analysis showed a similar level of c-Myc protein in one of the two P3HR1 infected tumors examined (in comparison to the AG876 virus infected and B95.8 infected tumors), although the other P3HR1 infected tumor expressed less c-Myc (**S6 Fig)**. Thus, loss of EBNA2 expression in P3HR1 tumors may be at least partially “rescued” by a compensatory EBNA2-independent c-Myc expression in some P3HR1 infected tumors.

### P3HR1 tumors are infiltrated with CD4 T cells

**Fig 11:**
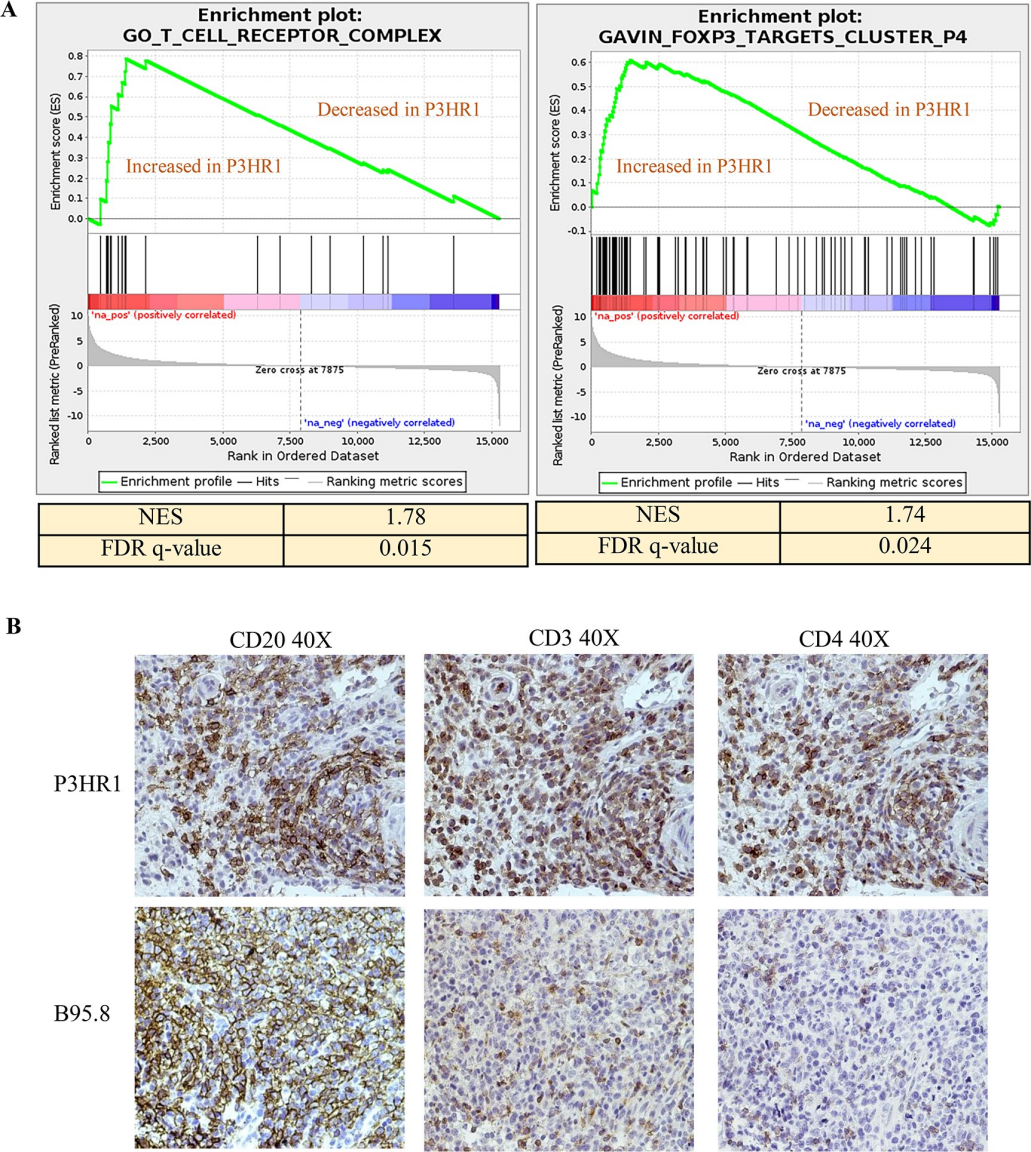
P3HR1 virus infected tumors are heavily infiltrated with CD4 positive T cells and show a T reg signature. **A.** Gene set enrichment analysis (GSEA) plot for the (“Go_T_cell receptor _complex”) and the “Gavin_foxp3-targets cluster _P4” gene set are shown in P3HR1 virus-induced lymphomas compared to B95.8 virus-infected lymphomas. **B**. IHC analysis using antibodies against CD20, CD3 or CD4 is shown in tumors infected with P3HR1 (mouse # 1 in **S1 Table**) or B95.8 viruses as indicated.

Consistent with the increased level of CD4 gene transcript in the P3HR1 tumors (**Fig 10**), GSEA analysis suggested that P3HR1 tumors have enrichment of genes consistent with an increased number of T cells (“Go_T_cell receptor _complex”), some of which are likely to be FoxP3 positive CD4+ T regs (“Gavin_FOXP3 _Targets _cluster-P4”) (**Fig 11A, Table A and B in S2 Table**). IHC analysis confirmed that P3HR1 tumors are heavily infiltrated by CD3+ and CD4+ T cells (**Fig 11B**).

### P3HR1 tumors have much more efficient antibody class switching compared to B95.8 tumors

**Fig 12:**
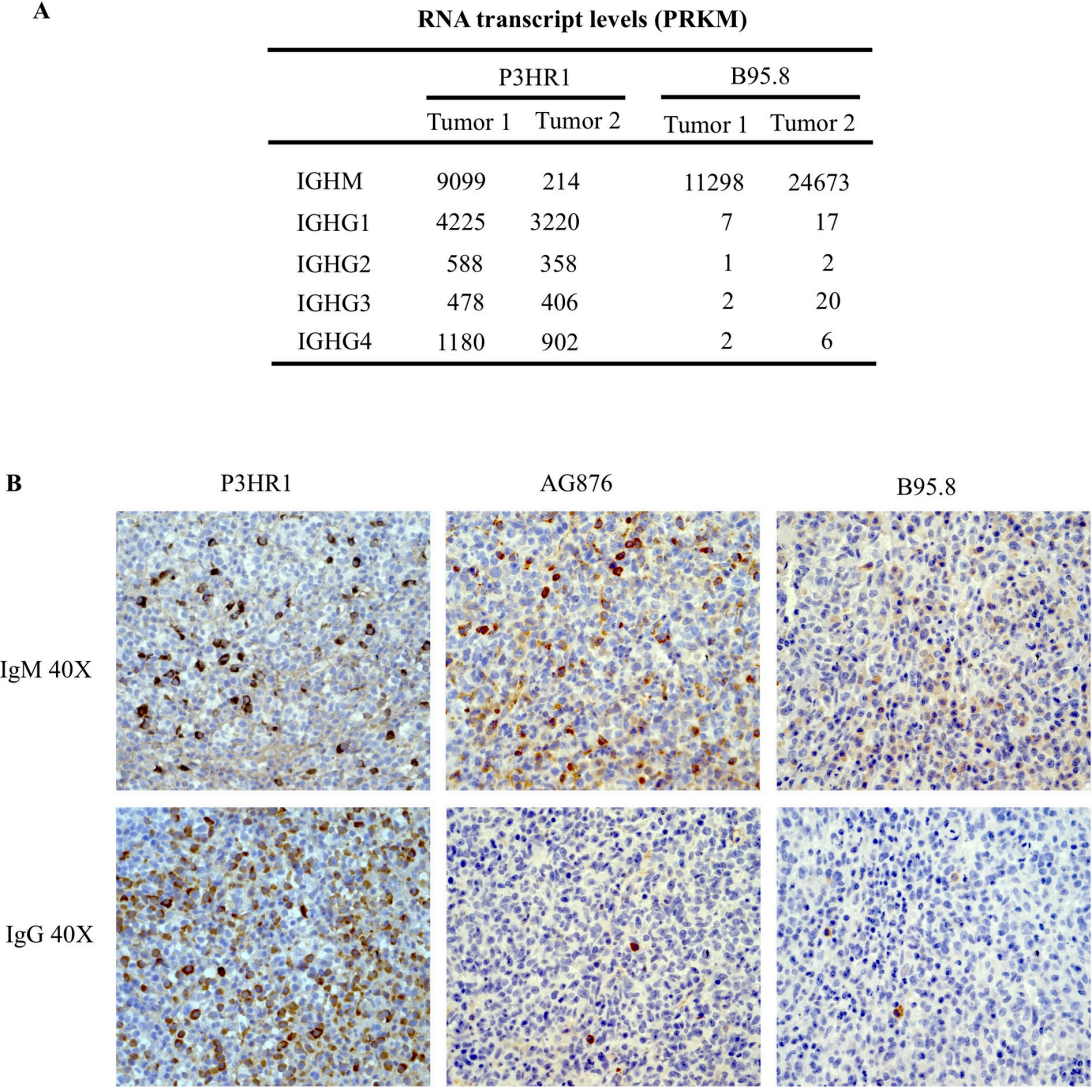
P3HR1 virus infected tumors express more IgG in comparison to AG876 and B95.8 virus infected tumors. **A**. The relative level of IGHM and various IGHG gene transcripts (as determined by RNAseq analysis) in P3HR1 virus-infected tumors, versus B95.8 virus-infected tumors is shown. Values are represented as PRKMs. **B.** Examples of IHC analysis using antibodies against IgM or IgG performed in tumors infected with P3HR1, AG876 or B95.8 viruses are shown. The P3HR1 tumor shown is derived from mouse #1 in **S1 Table**.

Interestingly, P3HR1 tumors also have much increased expression of IGHG genes compared to B95.8 tumors (**Fig 12A).** Increased expression of IgG in P3HR1 tumors versus AG876 or B95.8 tumors in CBH mice was confirmed by IHC (**Fig 12B**). Since cord blood B cells initially express only IgM, the increase in IgG expression in P3HR1 tumors suggests that EBNA2 inhibits IgM to IgG class switching in the CBH model. This is consistent with a previous report showing that EBNA2 inhibits AID expression [57], which is required for antibody class switching.

### P3HR1 infected lymphomas have a gene signature pattern suggestive of increased collagen

**Fig 13:**
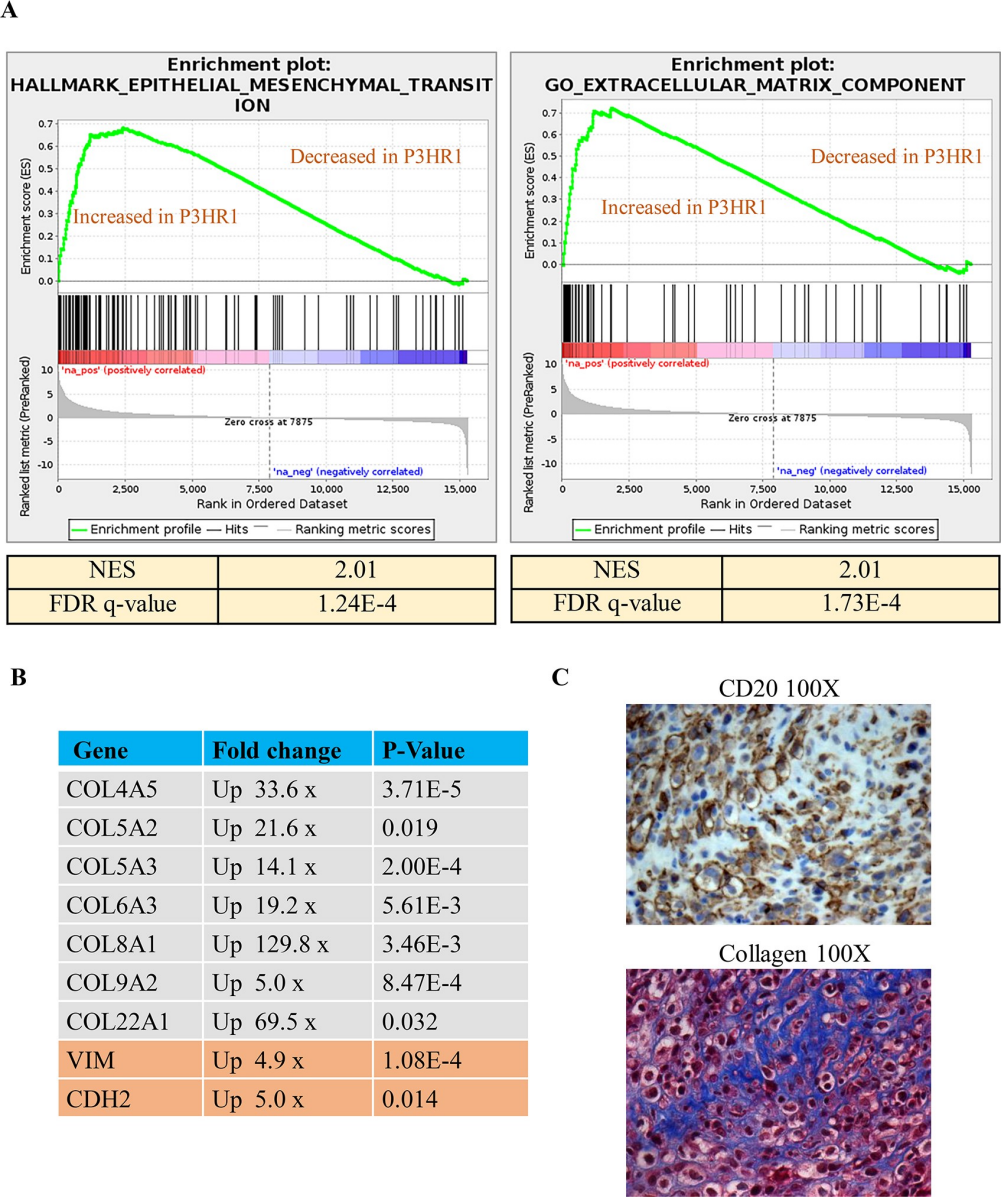
P3HR1 virus infected tumors have a gene signature pattern suggestive of EMT and are infiltrated with collagen. **A.** A gene set enrichment analysis (GSEA) plot for the “Hallmark_epithelial-mesenchymal_transition” and “Go extracellular matrix component” gene set are shown in P3HR1 virus-induced lymphomas compared to B95.8 virus-infected lymphomas. Normalized enrichment score (NES) and FDR q-value are shown to indicate strength of enrichment. A NES >1.6 and q-value < 0.05 were considered enriched. **B**. Expression levels of various human collagen genes and EMT markers (obtained from RNAseq data), along with the fold-upregulation in P3HR1 virus–infected versus B95.8 virus-infected cells (and the associated p-value) are shown. **C**. Masson’s trichrome staining (to detect collagen fibers in blue) and CD20 IHC staining (to detect lymphoma cells) was performed in a P3HR1 virus infected tumor derived from mouse #6 in **S1 Table**.

GSEA analysis also suggested that the “Hallmark_Epithelial_Mesenchymal_Transition” and “GO_Extracellular_Matrix” pathways (Fig 13A) are highly enriched (NES>1.6 and q-value<0.05) (**Fig 13A,Table C and D in S2 Table)** in P3HR1 virus-induced lymphomas compared to the B95.8 virus-induced lymphomas. A number of the differentially regulated genes in these two pathways proved to be human collagen genes and epithelial mesenchymal transition (EMT) marker genes (**Fig 13B**). Since it has been reported that collagen promotes survival of CHLs [41,42], we performed Masson’s trichrome staining to examine collagen levels in P3HR1-induced tumors. As shown in **Fig 13C,** P3HR1 tumors (like many human HLs) are heavily infiltrated with collagen. This result is consistent with the sclerosing CHL phenotype observed by H & E staining in one P3HR1 infected tumor in **Fig 6A**. Thus, high level collagen expression in the P3HR1 tumor environment may promote tumor cell growth in the absence of EBNA2 expression.

## Discussion

EBV-infected B cells are considered to be latently infected if the viral genome is replicated by the host machinery (using oriP) during S-phase. However, there are several different latency types that differ in the viral gene expression patterns and the ability to transform B cells *in vitro*. Type III latency (in which all 9 viral proteins are expressed) is characterized by expression of the viral EBNA2 transcription factor and is the only form of infection that can transform B cells *in vitro*. However, EBV-infected lymphomas in immunocompetent hosts (including CHL, DLBCL and BL) almost always have more stringent types of viral latency lacking EBNA2 expression, presumably because functional T cells easily recognize and eliminate cells supporting type III latency. To date, it has not been possible to model EBV-infected lymphomas lacking EBNA2 expression in the context of the intact viral genome. Here we show that a naturally occurring EBNA2-deleted EBV strain isolated from a human Burkitt lymphoma (P3HR1) causes lymphomas in the absence of EBNA2 expression in a cord blood-humanized mouse model. Furthermore, we demonstrate that some of the P3HR1-infected lymphoma cells sustain type II viral latency (LMP1-positive, EBNA2-negative, EBNA-LP-negative) and have phenotypic characteristics reminiscent of CHLs, whereas some P3HR1-infected lymphoma cells have the Wp-restricted form of viral latency (EBNA-2 negative, EBNA-LP-positive, LMP1-negative) that occurs in human BLs infected with P3HR1 and other P3HR1-like EBNA2 deleted EBV strains.

Our conclusion that the LMP1-positive/EBNA-LP-negative population of P3HR1-infected lymphoma cells have characteristics similar to the malignant Reed-Sternberg cells that define Hodgkin lymphomas is based upon the following: 1) this population of cells (like authentic human RS cells) expresses extremely high levels of CD30, and indeed, the level of CD30 in the P3HR1 cells with type II latency is much higher than the CD30 level observed in B95.8- and AG876-infected lymphomas that have type III latency (**Fig 5**); 2) this population of cells includes very large multinucleated cells that appear morphologically similar to RS cells (**Fig 6** and **S3 Fig**); 3) many of the large lymphoma cells in this population have lost CD45 expression (**Fig 6)**; and 4) a number of cellular genes reported to be activated in RS cells, including LGALS1 (galectin-1), CCL17 (TARC), CCL22, TNFRSF9 (CD137), and PDGFRA are expressed at much higher levels in the P3HR1-infected tumors compared to the B95.8 EBV-infected tumors (**Fig 10**). Our finding that LMP1-positive P3HR1 infected cells can upregulate expression of RS-associated factors is consistent with previous reports showing that LMP1 activates expression of a number of RS-associated cellular genes *in vitro* [39,40]. Interestingly, over-expression of LMP1 alone has also been shown to induce a multi-nucleated cell phenotype in EBV-negative BJAB lymphoma cells *in vitro* [59].

In contrast to previous *in vitro* studies that over-expressed LMP1 outside the context of the viral genome, our studies here demonstrate the ability of P3HR1 strain EBV to induce HL-like lymphomas in the context of viral infection. In addition to LMP1, human CHLs are thought to be driven by EBV LMP2A expression. LMP2A promotes survival of B cells by mimicking the effect of tonic BCR signaling, and this effect presumably compensates for loss of BCR expression that commonly occurs in human CHLs. Interestingly, we observed similar levels of LMP2A expression in the B95.8, AG876 and P3HR1 infected lymphomas (**Fig 8A**). At this point, the relative contribution of LMP1 versus LMP2A to the CHL-like phenotype in P3HR1 infected HL-like lymphomas remains undetermined.

Another characteristic of P3HR1-infected CBH mice reminiscent of human CHL lymphomas is the increased expression of the CD4 transcript (suggestive of CD4 T cell infiltration) and PD1 transcript (suggestive of exhausted T cells) in the P3HR1-infected lymphomas versus B95.8 infected lymphomas on RNAseq (**Fig 10**). Increased CD4 T cell infiltration of P3HR1 tumors was confirmed by IHC (**Fig 11B**). In addition, GSEA analysis suggested an enhanced signature of FoxP3-regulated genes (the key mediator of the Treg phenotype) in P3HR1 tumors (**Fig 11A**).

Clearly, however, the LMP1-high P3HR1 infected lymphoma cells do not perfectly mimic all aspects of human HL cells. For example, many of the cells still express some level of B cell markers, including CD20 (**S4 Fig**), whereas human CHL cells commonly lose these B cells markers. Thus, additional cellular gene mutations/epigenetic modifications may be required, in addition to P3HR1 virus infection, to more closely mimic human HL.

Another major unexpected and important finding from our studies here is the demonstration that Wp-restricted latency, a latency type so far only found in human BL tumors infected with naturally occurring EBNA2-deleted EBV variants, is also sufficient to induce EBV-infected DLBCL-like lymphomas in cord blood-humanized mice. This result is particularly surprising not only since the lymphoma cells with Wp-restricted latency in CBH mice are missing expression of the two major EBV oncoproteins, EBNA2 and LMP1, but also because both EBNA2 and LMP1 are required for the establishment of stable EBV infection of B cells *in vitro*. When full-length EBV infects B cells *in vitro*, the viral Wp is initially used to transcribe the EBNA-LP and EBNA2 genes [60,61]. EBNA2 then cooperates with EBNA-LP to transcriptionally activate the upstream Cp [61,62], inducing expression of all Cp-driven viral transcripts (including the three EBNA3 genes, EBNA1, and the viral BHRF1 microRNAs) [60,63] and also activates transcription of the divergent LMP1 and LMP2A promoters [64–67]. Transcription from the upstream Cp also inhibits the activity of the Wp [61], which is then furthered decreased by DNA methylation [68].

Our results here suggest that B cells infected with the EBNA2-deleted P3HR1 virus initially express the EBNA-LP, EBNA1 and EBNA3 genes via the Wp but do not activate LMP1 expression (via the viral Qp) unless the Wp is turned off. Exactly how Wp-restricted latency promotes DLBCLs in this model in the absence of LMP1 expression is not clear. Since Wp-restricted BL lines are more resistant to induction of apoptosis compared to BL lines with type I latency [21], one or more EBV proteins expressed in BL cells with Wp-restricted, but not type I, latency likely inhibits apoptosis The EBNA3A and EBNA3C proteins are obvious candidates as mediators of growth and survival in B cells with Wp-restricted latency, since both proteins are required for efficient transformation of B cells *in vitro*, and collaboratively EBNA3A and EBNA3C inhibit expression of the p16 tumor suppressor protein in EBV-infected LCLs [69]. We confirmed expression of EBNA3A by immunoblot in P3HR1-infected lymphomas in CBH mice (**Fig 8A**), although we could not confirm expression of EBNA3C due to the inability of commercially available antibody to recognize EBNA3C protein derived from type 2 EBV genomes. Interestingly, loss of expression of the BHFR1 protein, a BCL2 homologue that inhibits apoptosis, was previously shown to be more important for the continued survival of P3HR1-infected BL cells *in vitro* (which have Wp-restricted viral latency) than loss of the EBNA3C protein [22]. Thus, BHRF1 expression in the Wp-restricted P3HR1 infected lymphoma cells in CBH mice may also be important for initiation and/or growth of these lymphomas. Although we could not confirm expression of BHRF1 at the protein level in Wp-restricted P3HR1 infected lymphoma cells in CBH mice, this may reflect the relative insensitivity of commercially available BHRF1 antibody since Wp-restricted BL cells *in vitro* uniformly express BHRF1, and the RNA-seq results in **Fig 9** show a much higher level of BHRF1 transcript in the P3HR1 derived tumors compared to the B95.8 derived tumors.

The EBNA-LP protein may also contribute to the ability of P3HR1 to induce Wp-restricted lymphomas. EBNA-LP has been reported to function as a co-activator protein that collaborates with EBNA2 to activate certain viral promoters (including the LMP1 promoter) [70]. Interestingly, a recent study using an EBNA-LP deleted B95.8 EBV mutant reported that EBNA-LP is essential for the ability of EBV to transform human cord blood B cells *in vitro* although it is not absolutely required for the ability of EBV to transform adult peripheral blood B cells [71]; the exact reason for this phenotype is currently unknown. Of note, since the stereotypical EBNA2 deletion that occurs in BL tumors with Wp-restricted latency also deletes the c-terminal Y1 and Y2 exons of the EBNA-LP gene [21], it is possible that the smaller form of EBNA-LP expressed by P3HR1 virus and other similar EBNA2-deleted BL associated viral strains has acquired a *de novo* transforming function not present in the full length EBNA-LP protein. However, this seems unlikely given a previous report showing that a mutant EBNA-LP missing the carboxy terminal 45 amino acids is transformation defective *in vitro* [72].

Although our results here are the first to show that EBV can transform B cells in the absence of EBNA2 expression *in vivo*, it was previously demonstrated that over-expression of c-Myc can rescue the ability of EBV-transformed LCLs *in vitro* to survive and proliferate when EBNA2 expression is turned off [73]. Over-expression of translocated c-Myc in BL tumors may likewise allow the continued survival of BL tumors infected with EBNA2 deleted EBV. Interestingly, BL tumors (in patients) that have EBNA2-deleted EBV often contain both the full length virus and the deleted virus (although cell lines derived from these tumors often contain only the deleted virus), and sequence analysis suggests that the full-length and EBNA2-deleted viruses are derived from the same incoming viral genome [18–22]. Infection with full-length EBV first, as seems to occur in BL tumors prior to loss of EBNA2 expression, no doubt helps the virus to get established, and it is indeed quite remarkable that P3HR1 is able to establish persistent EBV infection in CBH mice in the absence of concomitant full-length EBV infection. Although we found that some P3HR1 infected lymphomas in CBH mice express less c-Myc compared to B95.8 infected lymphomas (**Fig 10A and S6 fig**), at least one P3HR1 infected tumor expressed a similar level of c-Myc (**S6 Fig**). Thus, a subset of P3HR1 infected lymphomas may select for c-Myc activation that occurs independently of EBNA2.

Another interesting and previously undescribed phenotypic difference in lymphomas infected with full length B95.8 and AG876 viruses, versus the P3HR1 virus, is the difference in the ability of infected cells to undergo antibody class switching from IgM to IgG (**Fig 12**). Since the B cells are derived from cord blood in this model, they presumably initially express IgM but little if any IgG. Our results suggest that the relative balance in latency protein expression in EBV-infected naïve B cells affects their ability to undergo class-switching. Both CD40 ligation (mimicked by LMP1) and AICDA expression (induced by EBNA3C) [74] strongly induce class-switching in normal B cells. However, EBNA2 has been reported to decrease expression of AICDA [57]. Since we found that both LMP1 high and LMP1 low cells commonly expressed IgG in P3HR1 infected CBH mice, loss of EBNA2 expression may be a dominant factor in allowing EBV-infected B cells to class switch from IgM to IgG usage in this model. Inhibition of class-switching by EBNA2 in newly EBV infected naïve B cells may protect virally infected cells from the DNA damage induced apoptosis that sometime occurs during the class-switching process.

Why P3HR1-infected cells expressing LMP1 are mutually exclusive with P3HR1-infected cells expressing EBNA-LP in the CBH model is not totally clear. We speculate that newly P3HR1-infected B cells initially assume Wp-restricted latency, since the Wp (which as activated by the B-cell specific factor, Pax5) is normally the first viral promoter used in B cells infected with wild-type B95.8 EBV [75]. DNA methylation of the Wp which occurs through poorly understood mechanism(s) as newly infected B cells transition from use of Wp to use of the EBNA2-depndent Cp [68], may likewise shut down use of Wp in a portion of P3HR1-infected cells and force selection for use of the alternative EBNA1 promoter, Qp (since continued expression of EBNA1 protein is required for replication of the viral genome through the oriP latency replication origin). Activation of LMP1/LMP2A expression in cells lacking EBNA2 expression presumably occurs via cellular transcription factors such as STATs and NF-κB that have been previously shown to activate the LMP1 promoter [9–11]. However, why the LMP1-expressing P3HR1-infected cells cannot also continue to use the Wp in order to express the EBNA-LP and the EBNA3 proteins is not clear, particularly since the combined expression of the LMP1/LMP2 proteins along with the EBNA3 and EBNA-LP proteins would presumably be more transforming than Wp-restricted or type II latency alone. Possibly a viral protein expressed in type II and/or type III latency (such as LMP1) acts to turn off expression of the Wp and/or results in its DNA methylation.

Another unanswered question is whether the ability of EBV to form tumors in the absence of EBNA2 expression is limited to viruses undergoing the particular stereotypical EBNA2 deletion observed in a subset of BL tumors; this EBNA2 deletion results in expression of the BHRF1 and EBNA3 genes from the Wp such that these proteins are still expressed [18–22]. Interestingly, in the absence of estradiol, we did not obtain tumors in NSG mice injected with a long-term LCL [76] that was immortalized with the combination of the P3HR1 virus and a plasmid expressing estrogen-inducible EBNA2. The inability of this cell line (which is maintained in the presence of EBNA2 expression and thus presumably uses the Cp to drive EBNA genes) to cause tumors suggests that differences in the viral gene expression pattern in newly P3HR1-infected B cells (such as the use of Wp instead of Cp) versus the long-term P3HR1-infected LCL are important for the ability of the P3HR1 virus to induce lymphomas in CBH mice. Since we have so far been unable to obtain tumors in CBH mice infected with an EBNA2-deleted B95.8 mutant that inhibits EBNA2 expression, but does not contain the stereotypical deletion that results in continued Wp activity and EBNA-LP/EBNA3/BHRF1 expression, we speculate that EBNA2-deleted EBV strains can only induce lymphomas in the context of the specific EBNA2 deletion found in Wp-restricted BLs.

In addition to the EBNA2-deleted viral genomes, Wp-restricted BL cell lines sometimes also contain defective/rearranged viral genomes (“W-Zhet”) in which the BZLF1 immediate-early protein is recombined next to the Wp latency promoter, resulting in constitutively active BZLF1 expression and high level lytic viral infection [77]. In our experiments here, in most cases we used P3HR1 virus derived from a P3HR1 BL cell clone that is lacking the W-Zhet genomes, making it unlikely that that these particles are required for P3HR1-mediated lymphomagenesis. Nevertheless, since we found that the P3HR1 induced tumors (particularly the EBNA-LP-high/LMP1-low foci) are highly lytic (consistent with our recent finding that type 2 EBV is much more lytic in B cells compared to type 1 EBV) [47], it is quite possible that lytic infection plays an important role in promoting the ability of P3HR1 to induce lymphomas in CBH mice.

Finally, whether EBNA2-deleted EBV variants, as typified by the P3HR1 strain used here, play an important role in the normal viral life cycle or whether they are merely “accidents” that nevertheless appear to contribute to EBV-positive BLs is not yet clear. Interestingly, the “W-Zhet” genomes that are sometimes associated with the EBNA2-deleted genomes can replicate and be horizontally transmitted to other cells, and have been reported by the Sixbey lab to be present in the saliva of normal individuals [78]. In addition, this same lab reported that approximately 30% of human CHL tumors carry W-Zhet genomes [79], although this result was not repeated by another group [80]. Currently, since relatively few EBV genomes have been sequenced from HL and DLBCL tumors, it is not clear whether infection with EBNA2-deleted EBV variants is selected for in a subset of human HL tumors, or DLBCLs, as it appears to be in human BLs. This will be an important are for future study.

## Materials and methods

### Ethics statement

All animal work experiments were approved by the University of Wisconsin-Madison Institutional Animal Care and Use Committee (IACUC) and conducted in accordance with the NIH Guide for the care and use of laboratory animals (protocol numbers M005197 and M005214). We anesthetized mice using isoflurane and euthanized animals by performing cervical dislocations on anesthetized mice.

### Cell lines and production of infectious EBV

The cell lines used to isolate P3HR1 virus are summarized in Supplemental S1 Table (**S1 Table**). In most cases P3HR1 strain virus (Cl 16) was isolated from the P3HR1 Burkitt lymphoma G668 (Cl 16) cell line (a gift from Dr. George Miller at Yale University via Dr. Bill Sugden, UW-Madison) [81]. In one case, virus isolated from the P3HR1 clone 5 cell line (which contains defective W-Zhet particles in addition to the EBNA2 deleted viral genome) was used to infect mice. All P3HR1 BL cell lines were maintained in RPMI 1640 medium supplemented with 10% fetal bovine serum (FBS), and 1% penicillin-streptomycin (pen-strep). To produce P3HR1 virus particles, BL cells were either treated with either 20 ng/ml phorbol-12-myristate-3-acetate (PMA) (Sigma) and 3mM sodium butyrate (Sigma), or stably infected with a retrovirus expressing the EBV BZLF1 protein fused to the hormone domain of the estrogen receptor (Z-HT), constructed as previously described [82] (a gift from Dr. Bill Sugden, University of Wisconsin-Madison) and treated with 200 nM of 4-HT (Sigma) for 72h. Viral particles harvested and concentrated as previously [47]. B95.8 strain virus was isolated from HEK293 cells latently infected with B95.8 strain EBV BACmid p2089, which expresses green fluorescent protein (GFP) and a hygromycin resistance gene as previously described [83]. EBV-infected HEK293 cells were maintained in Dulbecco modified Eagle medium (DMEM) supplemented with 10% fetal bovine serum (FBS), and 1% penicillin-streptomycin (pen-strep) and 100 ug of Hygromycin B. Infectious viral particles were produced from EBV-infected 293 cell lines by transfecting the cells with EBV BZLF1, BRLF1 and gp110 expression vectors as previously described [84]. AG876 virus was purified from AG876 Burkitt lymphoma cells (originally derived by Pizzo et al. [85]) stably infected with a retrovirus expressing the Z-HT fusion protein; cell lines were maintained in RPMI media with 10% FBS and 1 μg/ml puromycin. To stimulate AG876 virus production, ZHT-expressing cell lines were treated with 200 nM of 4-HT (Sigma) for 72h and viral particles harvested as previously described [47].

### EBV titer determination

B95.8 virus was tittered by infecting EBV-negative Akata cells with serial 10-fold dilutions of GFP-expressing B95.8 virus (similar to green fluorescent Raji cell titer assay previously described [83]). After 24 h, EBV-infected cells were treated with 20 ng/ml phorbol-12-myristate-3-acetate (PMA; Sigma) and 3 mM sodium butyrate (Sigma), and GFP-expressing cells were counted by fluorescence microscopy 24 h later. P3HR1 and AG876 virus titers were determined by comparing the relative EBNA1 protein expression induced by infection of EBV-negative Akata cells with 5-fold serial dilutions of each virus prep to that induced by serial 5-fold dilution of a known Akata (GFP-expressing) virus titer as previously described [47]. Cell lysates were collected after 72h and EBNA1 protein expression was quantitated by immunoblot analysis. Relative titer was calculated by comparing the relative EBNA1 protein expression in the GFP-expressing Akata virus infected cells with the known Akata virus titer. In three mice (#6, #7 and #8), the P3HR1 virus (**S1 Table**) used to infect mice was not tittered.

### Creation of EBV-infected cord blood-humanized NOD/LtSz-scid/IL2Rγnull mice

Immunodeficient NSG (NOD/LtSz-scid/IL2Rγnull) mice were purchased from Jackson labs (catalog number: 005557). Mononuclear cells (CBMCs) were isolated from fresh umbilical human cord blood (Umbilical Cord Blood Collection Program, UC Davis Health System) using Ficoll-Paque Premium solution (GE healthcare). CBMCs were infected with either P3HR1, B95.8 or AG876 viruses *in vitro* for 1.5 hours at 37°C. A minimum of 10 million cells were injected intraperitoneally into 6- to 10- week-old NSG mice which were age-matched. Mice were kept for a maximum of 92 days after injection of cord blood or were sacrificed if they become moribund prior to day 92. P3HR1 studies were performed using four different donors. In general, mice received 20,000 infectious units of the AG876 and B95.8 viruses (a dose sufficient to induce tumors in essentially 100% of infected animals [43,47]. Animals infected with the P3HR1 virus received 500,000 to 1 million infectious units; lower amounts of virus were not examined due to the expense of the model (and no difference was observed in the ability of different virus doses to cause tumors). P3HR1 was not tittered in three of the infected mice (**S1 Table**).

### Analysis of EBV infection and tumors

Following euthanasia, multiple different organs (including the lungs, spleen, pancreas, liver, gallbladder, mesenteric fat, and abdominal lymph nodes) were formalin fixed and then examined using a variety of techniques to determine if animals had persistent EBV infection and/or EBV-positive lymphomas and to assess the viral protein expression pattern. Samples from all EBV-infected animals were examined by H&E staining to determine if tumors were present and to assess the types of tumors in each animal. Tumors from EBV infected animals also underwent IHC staining using the antibodies listed in **Table 2**, as previously described [44,45]. Bone tissue was decalcified prior to paraffin embedding. Coauthor Erik A. Ranheim, a board-certified hematopathologist, performed the pathological analysis of the tumors.

**Table 2:** Antibodies used for immunohistochemistry (IHC) and western blot (WB).

Antibody	Manufacturer	Catalog number	Application
EBNA1	Santa Cruz Biotechnology	sc-81582	IHC & WB
EBNA2	Abcam Inc.	ab-90543	IHC& WB
EBNA3A	Exalpha	F115P	WB
EBNA-LP	Gift from Professor Yasush Kawagushi		IHC&WB
LMP1	Abcam Inc.	ab-7502	IHC
LMP1	Novus	NBP1-79009	IHC
LMP1	Abcam Inc.	ab-78113	WB
LMP2A	Santa Cruz Biotechnology	sc-101314	WB
LMP2A	Santa Cruz Biotechnology	sc-101315	IHC
BZLF1	Santa Cruz Biotechnology	sc-53904	IHC&WB
BZLF1	Santa Cruz Biotechnology	sc-17503	IHC
CD3	DakoCytomation	A0452	IHC
CD4	Leica Microsystems	NCL-L-CD4-368	IHC
CD8	Biocare	CRM311A	IHC
CD20	BD Pharmingen	BD555677	IHC
CD20	Novus	NBP1-30144	IHC
CD30	Novus	NBP2-49874	IHC
CD45	Abcam Inc.	ab-40763	IHC
CD137	Cell Signaling Technology	Cs-64594	IHC
CD138	Invitrogen	MA-12400	IHC
IRF4	Santa Cruz Biotechnology	sc-56713	IHC
IgG	Cell Marque	269A-15	IHC
IgM	Cell Marque	270A-15	IHC
c-Myc	Abcam Inc.	ab-32072	WB
PDGFR	Cell Signaling Technology	cs-5241	IHC

Antibodies used for IHC and immunoblot analysis are shown.

### Immunoblot analysis of tumor protein extracts

Frozen tumor samples were homogenized using the Covaris Cellcrusher tissue pulverizer system as per the manufacturer’s instructions. Immunoblotting was performed as described previously [86]. Briefly, tumor tissue was lysed in SUMO buffer plus protease inhibitors and then sonicated and centrifuged. Equivalent amounts of protein were separated in sodium dodecyl sulfate-10% or 8% polyacrylamide gel electrophoresis (SDS-PAGE) gels and transferred to nitrocellulose membranes. Membranes were blocked in 5% milk and then incubated with the appropriate primary antibodies diluted in 5% milk in 1X PBS and 0.1% Tween 20 (PBS-T). Membranes were then incubated with primary antibodies as listed in Table 2. After being washed, the membranes were incubated with the appropriate horseradish peroxidase-conjugated secondary antibodies (Pierce, Waltham, MA) in 5% milk–1X PBS-T for 1 h at room temperature and then washed again. Bound antibodies were visualized by use of enhanced chemiluminescent reagent (Pierce) according to the manufacturer’s instructions.

### RNA-seq analysis of tumor tissue

Analysis was performed as previously described [44]. RNA samples were harvested from frozen primary tumor tissue by homogenizing tissue with the Covaris Cryoprep Pulverizer as previously described [44]. Briefly, the pulverized tissue was then lysed in Trizol reagent (Invitrogen). RNA was isolated using phenol/chloroform extraction and total RNA was quantitated using a NanoDrop 2000 Spectrophotometer machine (ThermoFisher). RNA-seq libraries were prepared using the TruSeq Stranded mRNA Prep kit (illumina). Libraries were sequenced using an illumina HiSeq2500 platform at the University of Wisconsin Biotechnology Center DNA Sequencing Facility. RNA-seq data analysis was conducted by BioInfoRx (Madison, WI) as previously described [44]. Briefly, the fastQC program was used to verify raw data quality of the Illumina reads. The sequence data were mapped to human, mouse and virus genomes separately. The hg19 human genome and Ensembl gene annotations (v75), GRCm38 (mm10) mouse genome and Ensembl gene annotations (v82) were used for mapping. The raw sequence reads were mapped to the genome using Subjunc aligner from Subread [87]; then, we took the human and mouse alignment files (bam files) and assigned a read to one of the following categories (in the order listed below): 1. Mapped to both (mapping scores for both human and mouse >20, the difference between human and mouse mapping scores < = 10); 2. Mapped mostly to human (human mapping score>20, mapping to human genome better than to mouse genome (higher mapping score and fewer mismatches)); 3. Mapped mostly to mouse (mouse mapping score>20, mapping to mouse genome better than to human genome (higher score and fewer mismatches)); 4. Mapped to human (human mapping score minus mouse mapping score >10); 5. Mapped to mouse (mouse mapping score minus human mapping score >10); 6. No mapping (both mapping scores are 0); 7. Others. Reads from categories 2 and 4 were combined as human only reads and reads from categories 3 and 5 were combined as mouse only reads. These mouse only and human only alignment bam files were compared against corresponding gene annotation GFF files, and raw counts for each gene were generated using the featureCounts tool from Subread, with around 30–47% of reads overall assigned to human genes, and around 31–51% of reads overall assigned to mouse genes. The raw counts data were normalized using the TMM normalization method [88] in the program edgeR, and the normalized gene counts were transformed to log2 scale using the voom method from the R Limma package [89], then used for differential expression analysis. Functional interpretation of the differentially expressed genes was conducted based on GO terms, KEGG pathway and GSEA [90] methods.

### EBV genome analysis

EBV transcripts were analyzed as previously described [44]. Briefly, fastq files were aligned to an indexed B95-8 EBV genome using Burrows-Wheeler Aligner (BWA) [91]. SAMtools was used to generate sorted BAM files. A pileup of aligned reads was constructed as a Wig file using a python script.

### Immunoglobulin and T-cell receptor analysis

RPKMs for every IGHV and TRBV was determined using the RNA-seq analysis described above and was conducted by BioInfoRx (Madison, WI). We determined the total number of reads coming from all the IGHV or TRBV genes expressed in the tumor. We then determined the percentage of the total number of IGHV and TRBV reads for each IGHV and TRBV gene present in the tumor. Percentages were then plotted as pie charts in excel.

### Quantitative PCR analysis of tumor tissue

cDNA was reverse transcribed from RNA (same as that used in the RNAseq analysis) using random primers and GoScript reverse transcriptase (Promega). cDNA Real-time PCR was run by using SYBR green mix (Bio-Rad) in a Bio-Rad CFX96 machine. PCR running and data analysis were performed as previously described [44]. Primers used in qPCR are listed in Table 3. EBV primers were derived from Greijer et al. [49].

**Table 3:** Primers for qPCR analysis.

Gene	Forward Primer (5’-3’)	Reverse Primer (5’-3’)
Qp	GTGCGCTACCGGATGGC	CATGATTCACACTTAAAGGAGACGG
CCL22	GCGCGTGGTGAAACACTTCT	CCCTGAAGGTTAGCAACACCA
CD3	ATTGGCTGAGCAAGAAGGGA	ACAGCCACCTTGGAGGAAAC
CD4	CAAGGCTGGCAGTGACAGAA	AGCCTCTCTGAGCTCTGG
LGALS	GAGTGCCTTCGAGTGCGAGG	GGGTTGAAGTGCAGGCACAG
PDCD1	TCTCCGATGTGTTGGAGAAGC	GCGGCCAGGATGGTTCTT
PDGFA	GTATTCCACCTTGGCCACCTT	TGCAACACGAGCAGTGTCAA
PDGFRA	ATCCGGCGTTCCTGGTCTTA	GGTAATGAAAGCTGGCAGAGGA
GAPDH	TGCACCACCAACTGCTTAG	GATGCAGGGATGATGTTC
CD20	GCTGGCATCGTTGAGAATGAAT	TGCTGACAGGAGAACTATGTTAGAT

Sequences of primers used for qPCR assays are shown.

### Detection of collagen fibers by Masson’s trichrome staining

Masson’s trichrome stain kit was purchased from Polysciences LLP (Warrington, PA). Formalin-fixed, paraffin-embedded (FFPE) tissues were de-paraffinized in xylene (3 washes for 3 min each) and hydrated in graded ethanol to distilled water. Collagen fibers were then stained following the manufacturer’s protocol. After staining, collagen fibers were blue, cytoplasm and muscles were red and nuclei were black.

### Statistical analysis

All graphs were prepared in GraphPad Prism and Excel. Kaplan-Meier analysis was performed comparing tumor incidence over time (tumor incidence curve). The curve is inverted to show an increase in tumor development over time. The Log-rank test (two-sided) was performed on incidence curves and a p-value<0.05 was considered significant. Data were presented as mean ± standard error in bar graphs.

## Supporting information

S1 Fig (**A and B) P3HR1 infected tumors invaded multiple organs.** H & E staining of various P3HR1 infected tumors, invading different tissue types as indicated, is shown. Tumors are derived from mouse #1 #3, #5, #6, #7 in **S1 Table**.
(TIF)

S2 Fig P3HR1 infects B cells but not T cells in CBH mice. **A.** IHC co-staining was performed on a P3HR1 infected tumor using antibodies that detect EBNA1 or CD20 (mouse #7 in **S1 Table).** Red arrow shows examples of nuclear EBNA1 co-staining with cytoplasmic CD20 and blue arrow shows an example of a cell only staining with CD20. **B.** IHC co-staining was performed on a P3HR1 infected tumor using antibodies that detect EBNA1 or CD3 (mouse #7 in **S1 Table).** Blue arrow shows an example of a cell staining for CD3, and brown arrow shows an example of an EBNA1 staining cell.
(TIF)

S3 Fig P3HR1 infected tumors contain RS-like cells. H & E staining of P3HR1 infected tumor (derived from mouse #7 in S1 Table) is shown. A RS-like cell is indicated by black arrow.
(TIF)

S4 Fig P3HR1 infected RS-like cells have variable expression of CD20. IHC staining of a P3HR1 infected tumor derived from mouse # 2 in **S1 Table** was performed using a CD20 antibody. Examples of RS-like cells with high level CD20 staining (red arrows), medium staining (yellow arrows) and negative CD20 staining (white arrows) are shown.
(TIF)

S5 Fig P3HR1 and B95.8 infected lymphomas contain differentially expressed cellular genes. RNA was isolated from tumors infected with B95.8 or P3HR1 virus infected lymphomas, and RNA-seq performed. Mouse cell transcripts were removed from further analysis, and the levels of human genes in each tumor type was compared as described in the methods. The top 100 differentially expressed cellular genes in the RNA-seq analysis are shown above. The B95.8 and P3HR1 virus-induced lymphomas cluster together in a distinct pattern.
(PDF)

S6 Fig P3HR1 infected lymphomas express variable levels of c-Myc. Immunoblot analysis of proteins isolated from P3HR1 infected, AG876 infected or B95.8 virus infected lymphomas were performed using antibodies against c-Myc or tubulin as indicated. P3HR1 “1” protein is isolated from mouse #1 and P3HR1 “2” protein isolated from mouse #2 in **S1 Table**.
(TIF)

S1 Table P3HR1 virus source and dose in each infected mouse. (PDF)

S2 Table Gene profiles in listed GSEA plots. **A**. “Go_T_cell receptor _complex”. **B**. “Go_T_cell receptor _complex”. **C**. “Hallmark_epithelial-mesenchymal_transition”. **D**. “Go extracellular matrix component”.
(XLSX)
