# Supplementary figures and images for: EBNA2-deleted Epstein-Barr virus (EBV) isolate, P3HR1, causes Hodgkin-like lymphomas and diffuse large B cell lymphomas with type II and Wp-restricted latency types in humanized mice
Source: PLoS Pathog. 2020 Jun 15;16(6):e1008590. doi: 10.1371/journal.ppat.1008590 (PMC7316346; doi:10.1371/journal.ppat.1008590)

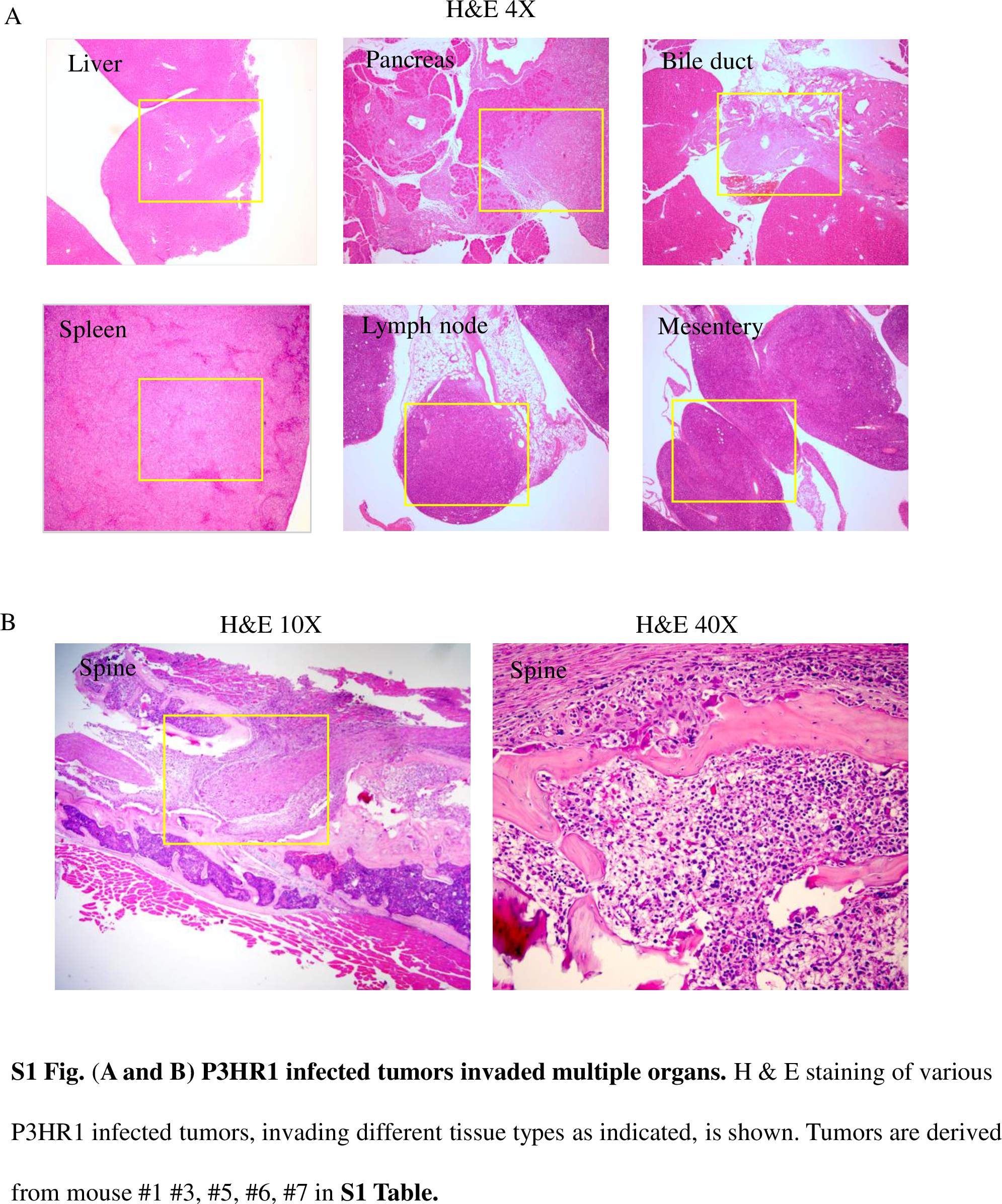

Supplement: S1 Fig — (A and B) P3HR1 infected tumors invaded multiple organs. H & E staining of various P3HR1 infected tumors, invading different tissue types as indicated, is shown. Tumors are derived from mouse #1 #3, #5, #6, #7 in S1 Table. (TIF) [file ppat.1008590.s001.tif]

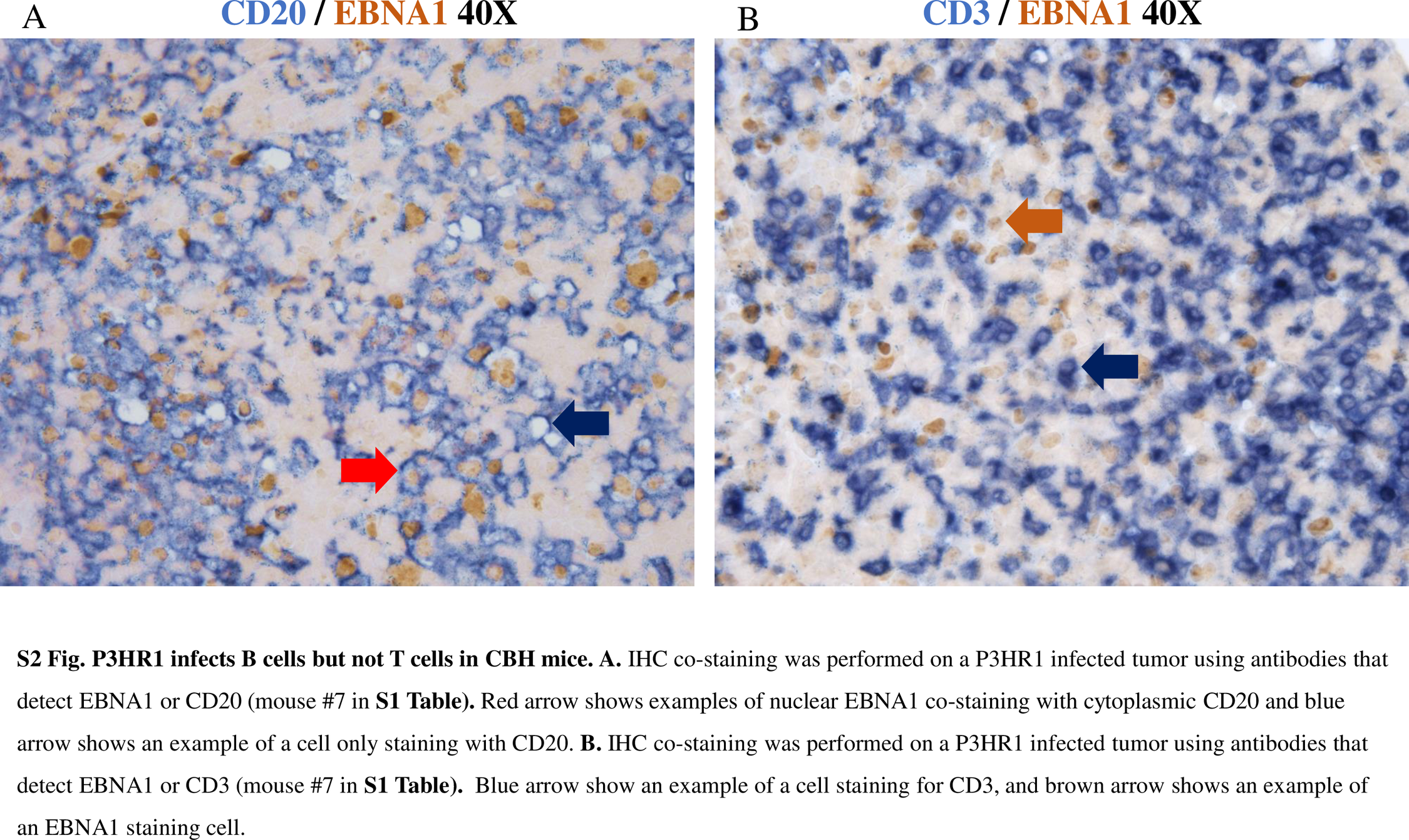

Supplement: S2 Fig — A. IHC co-staining was performed on a P3HR1 infected tumor using antibodies that detect EBNA1 or CD20 (mouse #7 in S1 Table). Red arrow shows examples of nuclear EBNA1 co-staining with cytoplasmic CD20 and blue arrow shows an example of a cell only staining with CD20. B. IHC co-staining was performed on a P3HR1 infected tumor using antibodies that detect EBNA1 or CD3 (mouse #7 in S1 Table). Blue arrow shows an example of a cell staining for CD3, and brown arrow shows an example of an EBNA1 staining cell. (TIF) [file ppat.1008590.s002.tif]

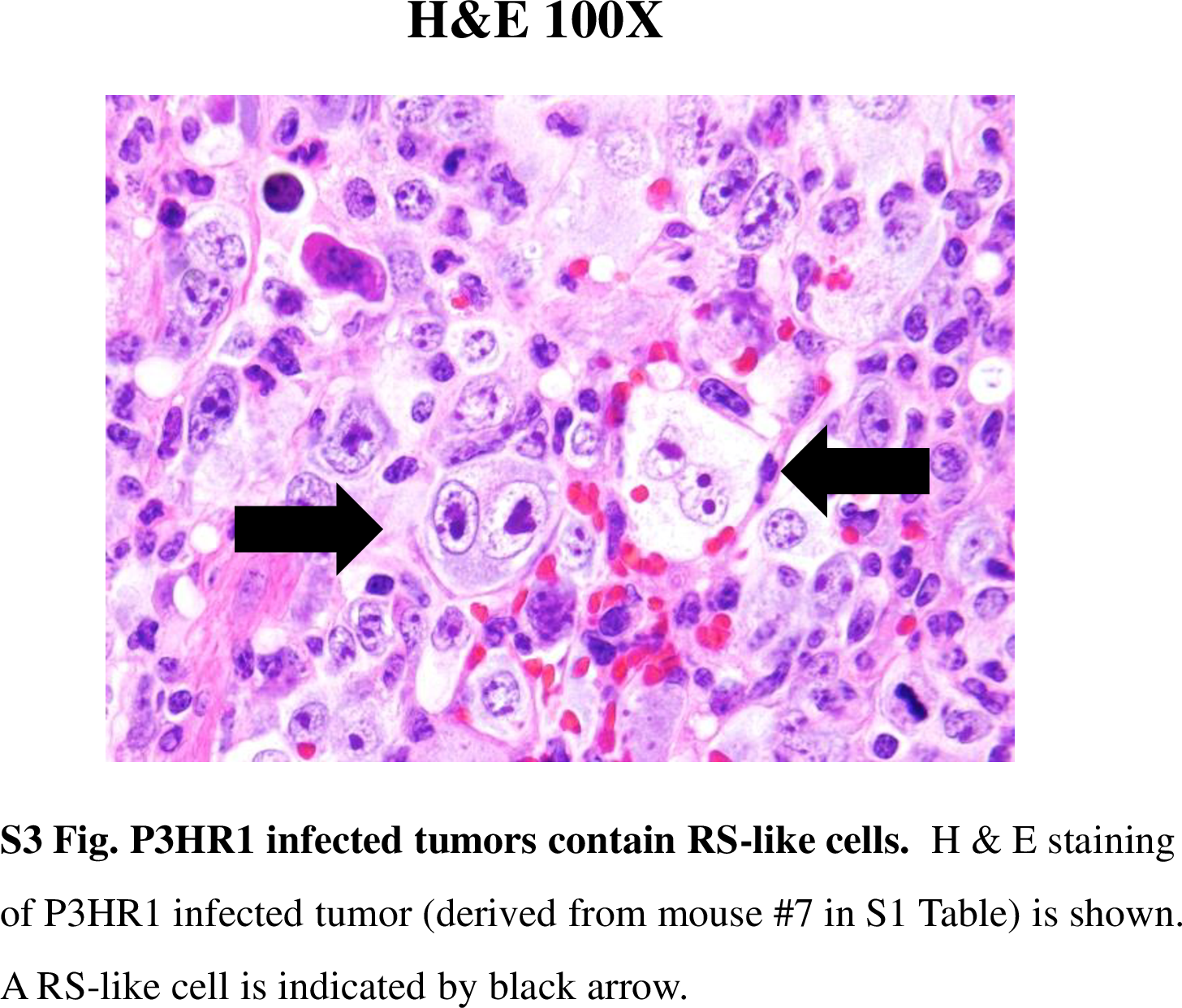

Supplement: S3 Fig — H & E staining of P3HR1 infected tumor (derived from mouse #7 in S1 Table) is shown. A RS-like cell is indicated by black arrow. (TIF) [file ppat.1008590.s003.tif]

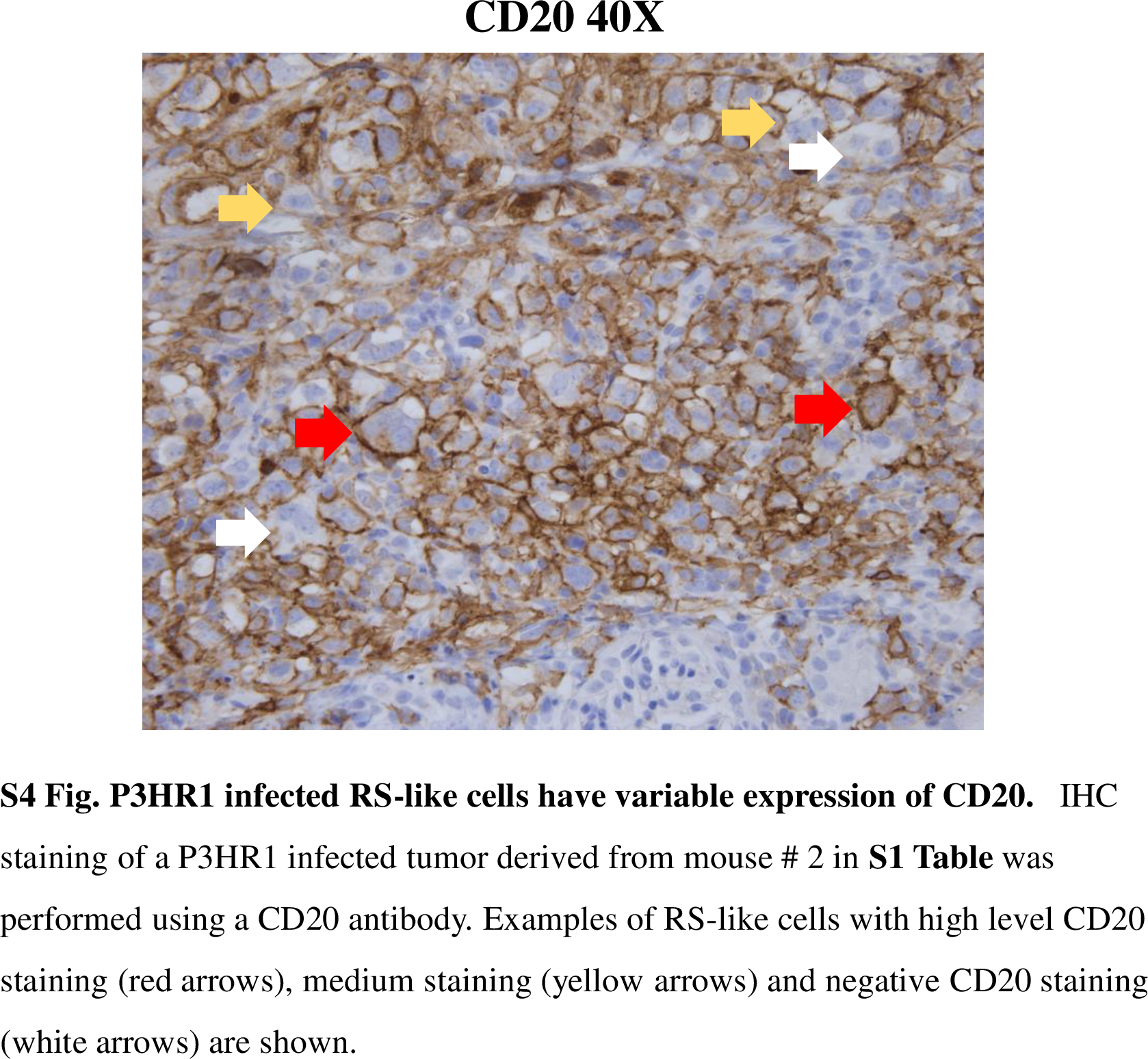

Supplement: S4 Fig — IHC staining of a P3HR1 infected tumor derived from mouse # 2 in S1 Table was performed using a CD20 antibody. Examples of RS-like cells with high level CD20 staining (red arrows), medium staining (yellow arrows) and negative CD20 staining (white arrows) are shown. (TIF) [file ppat.1008590.s004.tif]

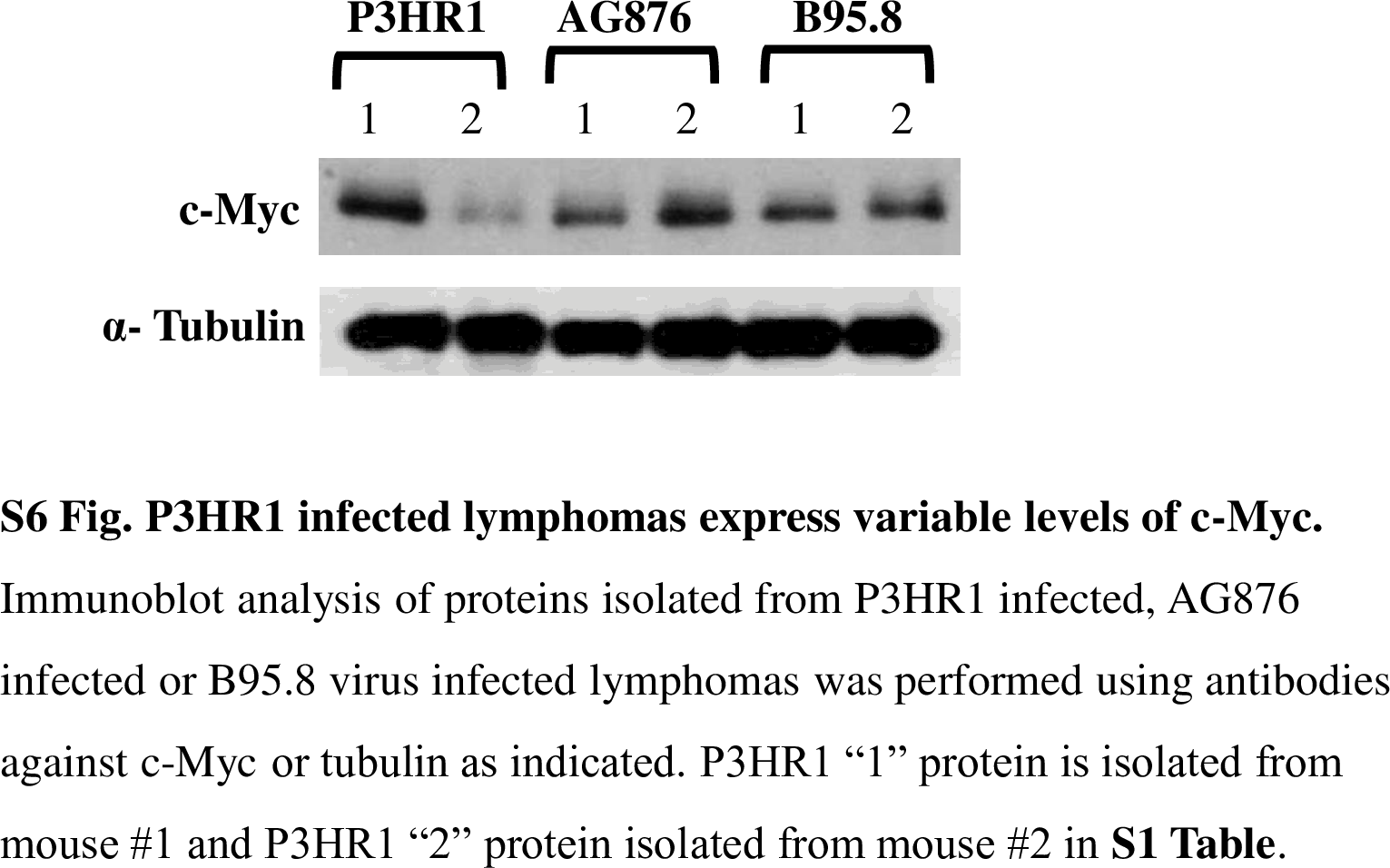

Supplement: S6 Fig — Immunoblot analysis of proteins isolated from P3HR1 infected, AG876 infected or B95.8 virus infected lymphomas were performed using antibodies against c-Myc or tubulin as indicated. P3HR1 “1” protein is isolated from mouse #1 and P3HR1 “2” protein isolated from mouse #2 in S1 Table. (TIF) [file ppat.1008590.s006.tif]
