# Supplementary material for: EBNA2-deleted Epstein-Barr virus (EBV) isolate, P3HR1, causes Hodgkin-like lymphomas and diffuse large B cell lymphomas with type II and Wp-restricted latency types in humanized mice
Source: PLoS Pathog. 2020 Jun 15;16(6):e1008590. doi: 10.1371/journal.ppat.1008590 (PMC7316346; doi:10.1371/journal.ppat.1008590)
Supplement: S5 Fig — RNA was isolated from tumors infected with B95.8 or P3HR1 virus infected lymphomas, and RNA-seq performed. Mouse cell transcripts were removed from further analysis, and the levels of human genes in each tumor type was compared as described in the methods. The top 100 differentially expressed cellular genes in the RNA-seq analysis are shown above. The B95.8 and P3HR1 virus-induced lymphomas cluster together in a distinct pattern. (PDF) [file ppat.1008590.s005.pdf]

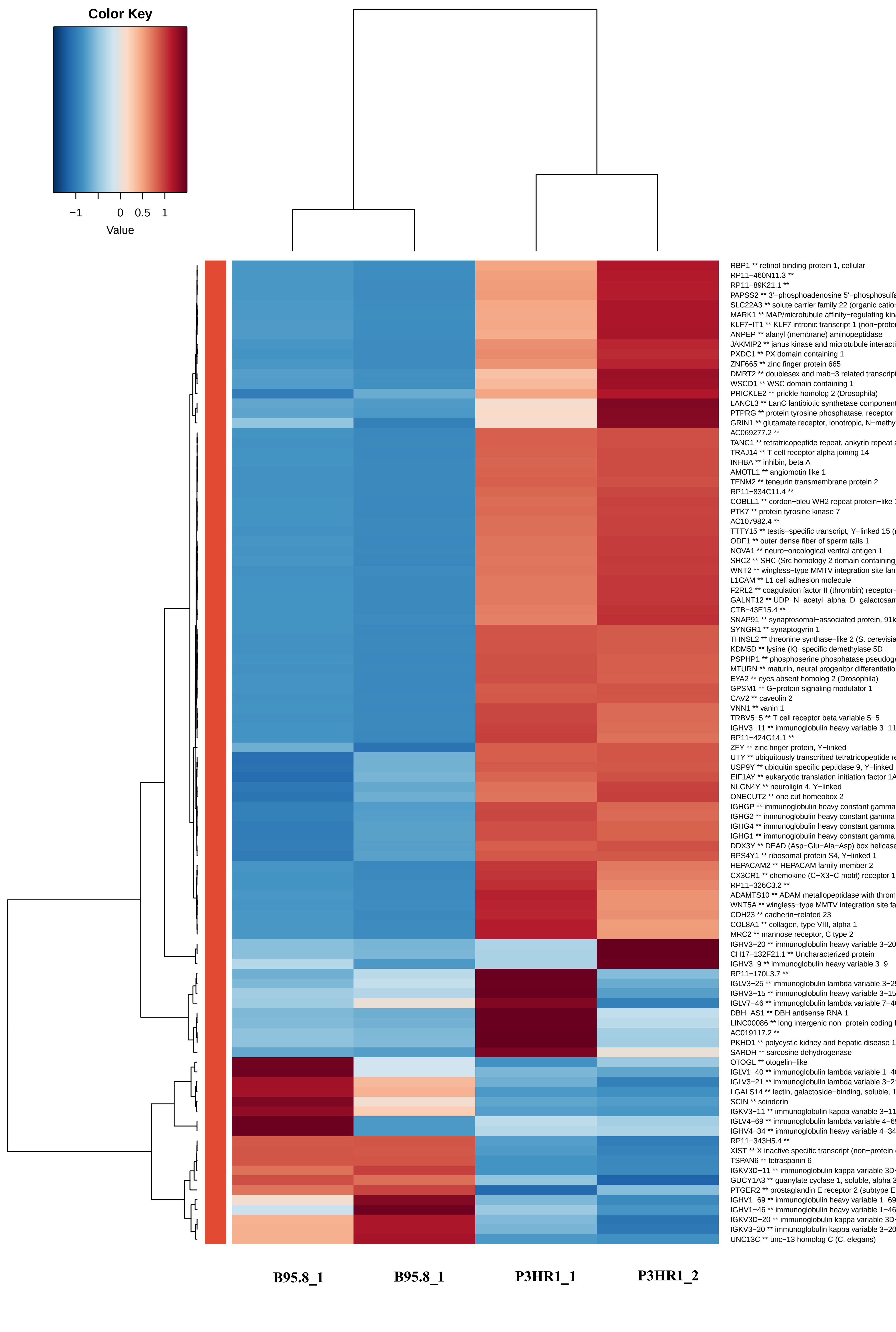
Color Key
−1
0
0.5
1
Value
RBP1 ** retinol binding protein 1, cellular
RP11−460N11.3 **
RP11−89K21.1 **
PAPSS2 ** 3'−phosphoadenosine 5'−phosphosulfa
SLC22A3 ** solute carrier family 22 (organic cation
MARK1 ** MAP/microtubule affinity−regulating kin
KLF7−IT1 ** KLF7 intronic transcript 1 (non−protei
ANPEP ** alanyl (membrane) aminopeptidase
JAKMIP2 ** janus kinase and microtubule interacti
PXDC1 ** PX domain containing 1
ZNF665 ** zinc finger protein 665
DMRT2 ** doublesex and mab−3 related transcript
WSCD1 ** WSC domain containing 1
PRICKLE2 ** prickle homolog 2 (Drosophila)
LANCL3 ** LanC lantibiotic synthetase component
PTPRG ** protein tyrosine phosphatase, receptor
GRIN1 ** glutamate receptor, ionotropic, N−methy
AC069277.2 **
TANC1 ** tetratricopeptide repeat, ankyrin repeat
TRAJ14 ** T cell receptor alpha joining 14
INHBA ** inhibin, beta A
AMOTL1 ** angiomotin like 1
TENM2 ** teneurin transmembrane protein 2
RP11−834C11.4 **
COBLL1 ** cordon−bleu WH2 repeat protein−like
PTK7 ** protein tyrosine kinase 7
AC107982.4 **
TTTY15 ** testis−specific transcript, Y−linked 15 (
ODF1 ** outer dense fiber of sperm tails 1
NOVA1 ** neuro−oncological ventral antigen 1
SHC2 ** SHC (Src homology 2 domain containing)
WNT2 ** wingless−type MMTV integration site fam
L1CAM ** L1 cell adhesion molecule
F2RL2 ** coagulation factor II (thrombin) receptor−
GALNT12 ** UDP−N−acetyl−alpha−D−galactosam
CTB−43E15.4 **
SNAP91 ** synaptosomal−associated protein, 91k
SYNGR1 ** synaptogyrin 1
THNSL2 ** threonine synthase−like 2 (S. cerevisia
KDM5D ** lysine (K)−specific demethylase 5D
PSPHP1 ** phosphoserine phosphatase pseudog
MTURN ** maturin, neural progenitor differentiatio
EYA2 ** eyes absent homolog 2 (Drosophila)
GPSM1 ** G−protein signaling modulator 1
CAV2 ** caveolin 2
VNN1 ** vanin 1
TRBV5−5 ** T cell receptor beta variable 5−5
IGHV3−11 ** immunoglobulin heavy variable 3−11
RP11−424G14.1 **
ZFY ** zinc finger protein, Y−linked
UTY ** ubiquitously transcribed tetratricopeptide r
USP9Y ** ubiquitin specific peptidase 9, Y−linked
EIF1AY ** eukaryotic translation initiation factor 1A
NLGN4Y ** neuroligin 4, Y−linked
ONECUT2 ** one cut homeobox 2
IGHGP ** immunoglobulin heavy constant gamma
IGHG2 ** immunoglobulin heavy constant gamma
IGHG4 ** immunoglobulin heavy constant gamma
IGHG1 ** immunoglobulin heavy constant gamma
DDX3Y ** DEAD (Asp−Glu−Ala−Asp) box helicas
RPS4Y1 ** ribosomal protein S4, Y−linked 1
HEPACAM2 ** HEPACAM family member 2
CX3CR1 ** chemokine (C−X3−C motif) receptor 1
RP11−326C3.2 **
ADAMTS10 ** ADAM metallopeptidase with throm
WNT5A ** wingless−type MMTV integration site fa
CDH23 ** cadherin−related 23
COL8A1 ** collagen, type VIII, alpha 1
MRC2 ** mannose receptor, C type 2
IGHV3−20 ** immunoglobulin heavy variable 3−20
CH17−132F21.1 ** Uncharacterized protein
IGHV3−9 ** immunoglobulin heavy variable 3−9
RP11−170L3.7 **
IGLV3−25 ** immunoglobulin lambda variable 3−2
IGHV3−15 ** immunoglobulin heavy variable 3−15
IGLV7−46 ** immunoglobulin lambda variable 7−4
DBH−AS1 ** DBH antisense RNA 1
LINC00086 ** long intergenic non−protein coding
AC019117.2 **
PKHD1 ** polycystic kidney and hepatic disease 1
SARDH ** sarcosine dehydrogenase
OTOGL ** otogelin−like
IGLV1−40 ** immunoglobulin lambda variable 1−4
IGLV3−21 ** immunoglobulin lambda variable 3−2
LGALS14 ** lectin, galactoside−binding, soluble, 1
SCIN ** scinderin
IGKV3−11 ** immunoglobulin kappa variable 3−11
IGLV4−69 ** immunoglobulin lambda variable 4−6
IGHV4−34 ** immunoglobulin heavy variable 4−34
RP11−343H5.4 **
XIST ** X inactive specific transcript (non−protein
TSPAN6 ** tetraspanin 6
IGKV3D−11 ** immunoglobulin kappa variable 3D−
GUCY1A3 ** guanylate cyclase 1, soluble, alpha 3
PTGER2 ** prostaglandin E receptor 2 (subtype E
IGHV1−69 ** immunoglobulin heavy variable 1−69
IGHV1−46 ** immunoglobulin heavy variable 1−46
IGKV3D−20 ** immunoglobulin kappa variable 3D−
IGKV3−20 ** immunoglobulin kappa variable 3−20
UNC13C ** unc−13 homolog C (C. elegans)
B95.8_1
B95.8_1
P3HR1_1
P3HR1_2
