# Supplementary material for: EBNA2-deleted Epstein-Barr virus (EBV) isolate, P3HR1, causes Hodgkin-like lymphomas and diffuse large B cell lymphomas with type II and Wp-restricted latency types in humanized mice
Source: PLoS Pathog. 2020 Jun 15;16(6):e1008590. doi: 10.1371/journal.ppat.1008590 (PMC7316346; doi:10.1371/journal.ppat.1008590)
Supplement: S1 Table — (PDF) [file ppat.1008590.s007.pdf]

S1 Table. P3HR1 virus source and dose in each infected mouse

Mouse #	Cell line to produce P3HR1 virus	P3HR1 virus dose
1	G668 (Cl. 16)	5X10^5 IU
2	G668 (Cl. 16)	5X10^5 IU
3	G668 (Cl. 16)	5X10^5 IU
4	G668 (Cl. 16)	5X10^5 IU
5	HH514-16	Unknown
6	HH543-5	Unknown
7	HH514-16	Unknown
8	G668 (Cl. 16)	1X10^6 IU
